# The Organization of Central Retinal Projections in Anna's Hummingbirds (*Calypte anna*) and Zebra Finches (*Taeniopygia castanotis*)

**DOI:** 10.1002/cne.70087

**Published:** 2025-09-05

**Authors:** Cristián Gutiérrez‐Ibáñez, Julia A. Bowen, Andrea H. Gaede, Douglas L. Altshuler, Douglas R. Wylie

**Affiliations:** ^1^ Department of Biological Sciences University of Alberta Edmonton Alberta Canada; ^2^ Department of Comparative Biomedical Sciences Royal Veterinary College London UK; ^3^ Department of Zoology University of British Columbia Vancouver British Columbia Canada

**Keywords:** avian, optic flow, optic tectum, retina, visual system

## Abstract

Hummingbirds (family *Trochilidae*) are easily recognized due to their unique ability to hover. Critical to hovering flight is head and body stabilization. In birds, stabilization during flight is mediated, among other things, by the detection of optic flow, the motion that occurs across the entire retina during self‐motion. Given this increased requirement for stabilization, it is not surprising that previous studies have shown that hummingbirds have neural specializations in the visual pathways involved in the detection of optic flow. Particularly, previous studies have found some structural and functional differences in the hummingbird brain, in the pretectal nucleus lentiformis mesencephali (LM): compared to other avian species, LM shows a massive hypertrophy, and LM neurons have unique response properties to optic flow stimuli. Here, we used intraocular injections of a neural tracer, cholera toxin subunit B (CTB) conjugated with a fluorescent molecule, to study the retinal projections in Anna's hummingbirds (*Calypte anna*) and compare them to those of a similarly sized non‐hovering species, the zebra finch (*Taeniopygia castanotis*). Retinal targets in both birds were similar and correspond closely to those reported in other birds from a variety of avian clades. Importantly, we found differences in the projections to LM between hummingbirds and zebra finches. Consistent with previous reports of specialization of LM, it was more intensely labelled compared to other retinal‐recipient nuclei in hummingbirds. Moreover, this increase in intensity was most apparent in the lateral subnucleus. This study reinforces previous evidence that the LM of hummingbirds is adapted to sustain the unique flight abilities of this clade.

## Graphical abstract

Retinal projections in the Anna's hummingbirds (*Calypte anna*) where studied using intraocular injections of a neural tracer and compared to those of a non‐hovering species, the Zebra finch (*Taeniopygia castanotis*). Retinal targets in both birds were similar and correspond closely to those reported in other birds from a variety of avian clades. Differences were found in the projections to LM between hummingbirds and zebra finches, which is consistent with previous reports of specialization of LM in hummingbirds.

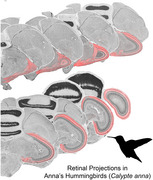

**Abbreviations**
AOS: accessory optic system
AP: area pretectalis
APd: area pretectalis, pars dorsalis
APTD: anterior pretectal nucleus, dorsal part
APTp: anterior pretectal nucleus, superficial cell plate
APTs: anterior pretectal nucleus, superficial plexiform layer
APTV: anterior pretectal nucleus, ventral part
CO: optic chiasm
DLAmc: n. dorsolateralis anterior thalami, pars magnocellularis
DLL: dorsolateralis anterior thalami, pars lateralis
ECR: ectopic cell region
GLv: eniculatus lateralis, pars ventralis
GTc: caudal tectal gray
GTr: rostral tectal gray
IGL: intergeniculate leaflet
ION: isthmo‐optic nucleus
LA: n. lateralis anterior thalami
LdOPT: lateralis dorsalis optici principalis thalami
LM: lentiformis mesencephali
LMl: lentiformis mesencephali, pars lateralis
LMm: lentiformis mesencephali, pars medialis
LTC: lateral terminal nucleus, commisural part
LTJ: lateral terminal nucleus, juxtacommisural part
MT: medial terminal nucleus
nBOR: n. of the basal optic root
nMOT: marginalis tractus optici
OT: optic tract
PG: pregeniculate nucleus
PT: pretectalis
RGC: retinal ganglion cell
Rt: rotundus
SO: stratum opticum
SpRt: suprarotundus
TeO: optic tectum
TGS: superficial tectal gray
TIO: isthmo‐optic tract
VLT: ventrolateralis thalami
vSCN: visual suprachiasmatic nucleus

## 1 Introduction

In all vertebrates, the retina targets multiple brain areas, thus giving rise to parallel visual pathways to the brain (Ebbesson 1970; Riss and Jakway 1970; Hodos and Butler 2008). This includes targets in the thalamus, hypothalamus, pretectum, and mesencephalon (Ebbesson 1970). It is largely acknowledged that in all vertebrates, distinct visual pathways subserve different functions (Rodieck et al. 1993). Examples of this functional specialization include the visual pathways that detect optic flow, the motion that occurs across the entire retina during self‐motion (Gibson 1954), which are highly conserved among all vertebrates. These visual pathways usually include two targets, the accessory optic system in the mesencephalon and at least one target in the pretectum (Simpson 1984; Fite 1985; McKenna and Wallman 1985; Weber 1985; Giolli et al. 2005; Gamlin 2006). In birds, these nuclei are the nucleus of the basal optic root (nBOR) of the accessory optic system (Karten et al. 1977; Brecha et al. 1980) and the pretectal nucleus lentiformis mesencephali (LM, Gamlin and Cohen 1988). In birds, as in all vertebrates, these pathways are involved in the stabilization of the retina through the generation of the optokinetic response (Simpson 1984; Waespe and Henn 1987; Giolli et al. 2005; Gamlin 2006). Retinal image stabilization is crucial for all animals, as both visual acuity and relative velocity discrimination are impaired during movement of the retina (Westheimer and McKee 1975; Nakayama 1981).

Among birds, hummingbirds (Family *Trochilidae*) are easily recognized due to their unique ability to sustain hovering flight (Altshuler and Dudley 2002). Critical to hovering flight is head and body stabilization. To guide their beak into flowers, hummingbirds must maintain a stable position in time and space, even in the presence of environmental perturbations, like wind gusts. Stabilization during flight is mediated by several vestibular, visual, and proprioceptive reflexes, including the above‐mentioned optokinetic response (Wilson and Jones 1979; Horowitz et al. 2004; Gutiérrez‐Ibáñez et al. 2023). Given this increased requirement for stabilization, it is not surprising that previous studies have shown that hummingbirds have neural specializations in the visual pathways involved in the detection of optic flow, which drives the optokinetic response. For example, Iwaniuk and Wylie (2007) showed that hummingbirds have an LM (but not an nBOR) that shows a massive hypertrophy compared to those of other birds. Furthermore, LM neurons in hummingbirds have unique tuning properties compared to other birds. First, whereas in all other tetrapods LM neurons show a strong bias for optic flow moving in the temporal‐to‐nasal direction (Hoffmann and Schoppmann 1981; Fite 1985; McKenna and Wallman 1985; Mustari and Fuchs 1990; Wylie and Crowder 2000; Ibbotson and Price 2001), such a bias is much less pronounced in hummingbirds (Gaede et al. 2017; Smyth et al. 2022). Second, hummingbird LM neurons are tightly tuned to faster stimulus velocities and low spatial frequency compared with other birds (Winterson and Brauth 1985; Wylie and Crowder 2000; Gaede et al. 2022; Smyth et al. 2022).

Given the above specializations in LM and the particular flight behavior of hummingbirds, other specializations may exist concerning LM and perhaps other parts of the visual system. One possibility is that differences exist in the projection patterns from the retina to the brain. While in most birds, the retina targets the same nuclei, some differences exist. For example, in many birds, the retina projects only to the superficial layers (1–7) of the optic tectum (TeO; Hunt and Webster 1975; Norren and Silver 1989; Shimizu et al. 1994), but in the Chilean Tinamou (*Nothoprocta perdicaria*) and the Banded Nightjar (*Systellura longirostris*), projections to deeper layers have been found (Krabichler et al. 2015; Salazar 2023). To date, retinal projections have not been studied in hummingbirds.

**FIGURE 1:**
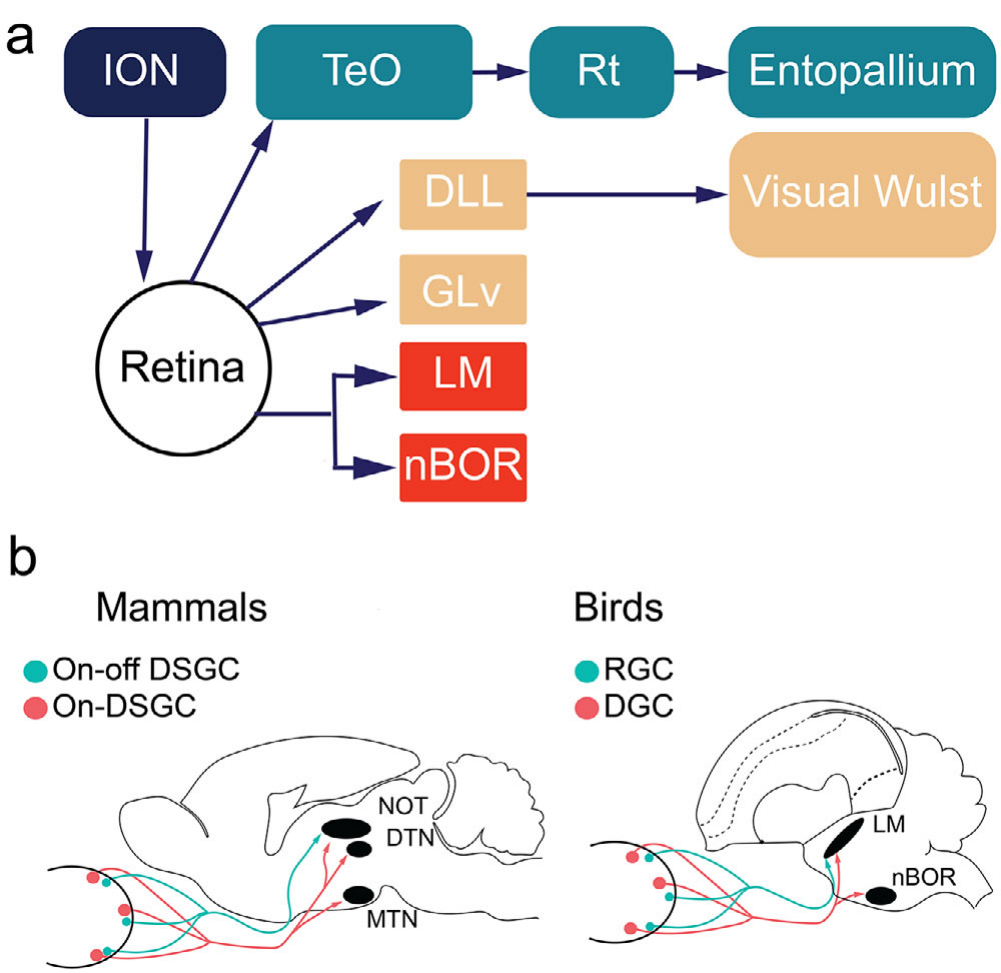
Visual pathways in birds. (a) A reduced schematic shows the three major visual pathways in birds. The tectofugal pathway is shown in green, and the thalamofugal pathway is shown in yellow. (b) Two schematics compare the retinal inputs to the accessory optic system (AOS) and the pretectum in birds and mammals. In birds, displaced ganglion cells (DGCs) project to the nucleus of the basal optic root (nBOR), part of the AOS, and to the pretectal nucleus lentiformis mesencephali (LM). Additionally, LM receives projections from some regular retinal ganglion cells (RGCs). In mammals, the medial and dorsal terminal nuclei (MTN and DTN, respectively), which are part of the AOS, and the pretectal nucleus of the optic tract (NOT), receive projections from a specific type of direction‐selective RGCs (DSGCs). NOT additionally receives projections from a second type of retinal ganglion cells, ON–OFF DSGCs (Dhande et al. 2013). AOS accessory optic system; dLGN dorsal lateral geniculate nucleus; LM nucleus lentiformis mesencephali; MTN medial terminal nucleus; nBOR nucleus of the basal optic root; NOT nucleus of the optic tract.

In birds, retinal ganglion cells reach several targets in the hypothalamus, pretectum, thalamus, and midbrain. Figure 1 shows a schematic of the main visual pathways in birds. About 90% of retinal axons project to the TeO in the midbrain (Mpodozis et al. 1995), which gives rise to several pathways (Reiner and Karten 1982; Luksch 2003; Wylie et al. 2009), including the ascending tectofugal pathway to the nucleus rotundus (Rt) in the thalamus, which then projects to the entopallium in the forebrain (Karten and Hodos 1970; Benowitz and Karten 1976; Krützfeldt and Wild 2004; Fredes et al. 2010). A second target is the dorsal thalamus, where a group of nuclei collectively referred to as the nucleus opticus principalis thalami (OPT) receives retinal projections (Miceli et al. 1975; Remy and Güntürkün 1991). These thalamic nuclei project to the visual Wulst (Karten et al. 1973). Other retinal‐recipient targets include the ventral geniculate nucleus (GLv), as well as the LM in the pretectum and nBOR in the midbrain. Additionally, there is a retinal projection to the suprachiasmatic nuclei in the hypothalamus (Norgren and Silver 1989) and several other nuclei in the pretectum (Gamlin and Cohen 1988). Finally, in birds, there is a substantial centrifugal projection to the retina arising from the isthmo‐optic nucleus (ION, Figure 1; Woodson et al. 1995; Repérant et al. 2006). Given the multiple targets and varied functions of the different visual pathways, it is possible that hummingbirds present differences in any of them when compared to other birds, but given the anatomical and functional differences described in LM, this is the most likely area.

In birds (and other vertebrates, but not mammals), a subset of ganglion cells can be found in the amacrine cell layer rather than the ganglion cell layer, which are called displaced ganglion cells (DGCs; Karten et al. 1977; Fite et al. 1981; Reiner 1981; Bellintani‐Guardia and Ott 2002; Wylie et al. 2014; Gutierrez‐Ibanez et al. 2018). DGCs project largely to nBOR, but recent studies have shown that in pigeons, zebra finches (ZFs), and hummingbirds, LM also receives projections from DGCs, in addition to projections from “traditional” ganglion cells (Figure 1b; Wylie et al. 2014; Gutierrez‐Ibanez et al. 2018). Based on similar projections to the accessory optic system, the DGCs of birds are likely homologous to the ON‐DSGCs of mammals, which are direction sensitive (Dhande et al. 2013; Gutierrez‐Ibanez et al. 2018). Given the specialization in hovering and the reliance on optic flow during hovering flight of hummingbirds (Altshuler and Dudley 2002; Dakin et al. 2016; Ros and Biewener 2016; Baliga et al. 2024), the projections of DGCs, in particular, may be different in this group. Here, we used intraocular injections of a neural tracer, cholera toxin subunit B (CTB) conjugated with a fluorescent molecule, to study the retinal projections in Anna's hummingbirds (AH, *Calypte anna*) and compared them to those of a similar‐sized but non‐hovering species, the ZF (*Taeniopygia castanotis)*.

## 2 Methods

### 2.1 Animals

All experimental procedures were approved by the University of British Columbia Animal Care Committee per the guidelines set out by the Canadian Council on Animal Care. Experiments were performed on two adult AHs (*C. anna*) caught on the University of British Columbia campus and two adult male ZFs (*T. castanotis)* purchased from a local breeder.

### 2.2 Surgery and Tracer Injection

Surgeries were performed using a custom‐built stereotaxic frame designed for small bird neurosurgery with integrated gas delivery (David Kopf Instruments, CA, USA). Birds were anesthetized with 1.2% isoflurane mixed with oxygen through a fitted mask. Intermittent doses of 0.3%–0.7% isoflurane were delivered as necessary to maintain the anesthetized state. To inject the eye, a small incision was made on the dorsal surface of the eye, behind the scleral ring. An ophthalmoscope was used to guide the tip of a Hamilton syringe behind the vitreous humor and as close to the retina as possible. The syringe was filled with 10% cholera toxin B (CTB)‐AlexaFluor 594 (Thermo Fisher Scientific, C34777) in phosphate‐buffered saline (PBS) with 2% dimethylsulfoxide. Several small injections were made in different locations until 10–15 µL of tracer was injected. The diffusion of the tracer was confirmed with an ophthalmoscope, and then the skin was closed with cyanoacrylate (Vetbond, 3 M), and the animals were given buprenorphine (0.012 mg/kg im) as an analgesic.

### 2.3 Brain Extraction and Sectioning

After a recovery period of 3–4 days, birds were deeply anesthetized (ketamine/xylazine mixture im) and transcardially perfused with saline (0.9% NaCl) and 4% paraformaldehyde in 0.1 M PBS (pH 7.4). Brains were extracted and immersed in paraformaldehyde for at least 24 h. Subsequently, brains were cryoprotected in 30% sucrose in 0.01 M PBS (pH 7.4). Next, the brains were embedded in gelatin and cryoprotected in 30% sucrose in PBS overnight. Using a sliding microtome, brains were sectioned into two series in the coronal plane at a thickness of 40 µm. The sections were then mounted on gelatinized glass slides, dried, and stored at 4°C.

### 2.4 Microscopy and Image Analysis

Sections were examined and photographed using a Leica microscope (Leica DM6 B, Leica Microsystems, IL) containing TX2 (red), L5 (green), and DAPI (blue) fluorescence filters. Images were captured using a Leica K5 camera and Leica Application Suite X (LAS X, RRID: SCR_013673) software. Only for images used in figures that show the fluorescence labeling were the images enhanced (e.g., brightness, saturation) using Adobe Photoshop (RRID: SCR_014199). After acquiring all fluorescent images, we removed the coverslips and dried the slides for storage or subsequent Nissl staining with thionin. In short, slides were air dried, hydrated through a graded series of ethanol solutions, stained for 3 min in a 0.2% thionin solution in a 4.4 pH acetate buffer, then dehydrated through a graded series of ethanol solutions, cleared in Citrasolv (Fisher Scientific), and coverslipped in Permount (Fisher Scientific). Images were then taken of the Nissl‐stained sections with the same microscope as above, and these were overlaid upon the fluorescent images of the same sections. This allowed for an accurate delineation of the borders of brain nuclei that received retinal projections.

The intensity of fluorescent labeling was measured using Leica Application Suite X (LAS X, RRID: SCR_013673). Intensity was measured in fluorescent microphotographs of the coronal sections for each case, which were all taken using the same light intensity and exposure time. Intensity was measured using a tool that measures intensity across a straight line, that is, in one dimension. In some instances, intensity was measured in a representative line encompassing different retinorecipient nuclei or tectal layers, as shown in the corresponding figure. Intensity was standardized and centered within each line.

Additionally, to compare the average labeling intensity across the whole nuclei, in one series of each species, we measured labeling intensity throughout the anteroposterior extent of the medial and lateral subdivisions of LM (LMl and LMm, respectively), GLv, GT, and nBOR. In this case, we measured intensity using several different lines, one for each nucleus of interest. Each line was always aligned with the longest possible axis of the nuclei to maximize the number of measured points. In the latter case, the intensity was standardized and centered within each species. We performed an ANOVA with Tukey post hoc comparisons using the stats package in R (R Core Team 2022) to test for differences in average intensity between the different nuclei measured only within a species and case.

### 2.5 Nomenclature

For consistency with our previous work in the visual nuclei of ZFs and hummingbirds (e.g., Gutierrez‐Ibanez et al. 2018; Gaede et al. 2019, 2024; Wylie et al. 2023) we have kept the nomenclature unchanged. This nomenclature is based on Gamlin and Cohen's (1988) description of retinal projections to the pretectum of the pigeon. The only difference is that we have used the abbreviations GTr and GTc to refer to the rostral and caudal subdivisions of the tectal gray (GT) and omitted the use of dorsal and central subdivisions of GT as in Gamlin and Cohen 1988. Puelles et al. (2018) have recently proposed a different nomenclature based on proposed homologies with the mammalian pretectum nuclei. Table 1 shows this nomenclature.

**TABLE 1:** Comparison of nomenclature for retinorecipient and adjacent nuclei in the pretectum and accessory optic system of birds by different authors. For abbreviation, see the list.

This manuscript	Karten et al. (1967) (Pigeon)	Gamlin and Cohen (1988) (Pigeon)	Puelles et al. (2018) (Chicken)
LMl	LMpc	LMl	LTJ/LTC
LMm	LMmc	LMl	APTs
LPC		LPC	APTp
PPC	PPC	PPC	APTD/APTV
GTr		GTd/GTv	TGS
GTc		GTc	TGS
nBOR	nBOR	nBOR	MT
GLv	GLv	GLv	PG

## 3 Results

### 3.1 Primary Visual Projections

**FIGURE 2:**
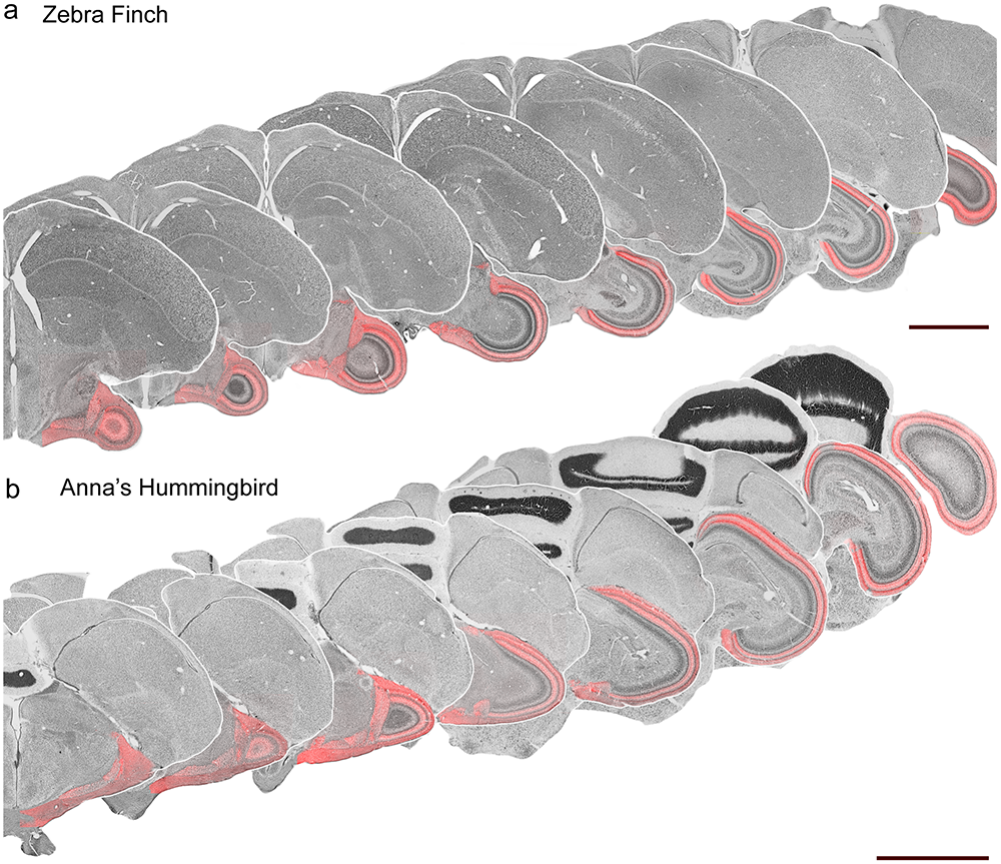
Retinal projections to the optic tectum in the zebra finch and the Anna's hummingbird. (a, b) show the projection pattern of retinal terminals in the contralateral thalamus and optic tectum of the zebra finch and the Anna's hummingbird, respectively. The retinal projections labeled with fluorescent conjugated cholera toxin subunit B are superimposed over Nissl‐stained sections. The separation between sections ranges from 240 to 480 µm. Importantly, the entire TeO is labeled, which confirms that the tracer was taken up by the whole extent of the retina in both species. Scale bars = 2 mm.

**FIGURE 3:**
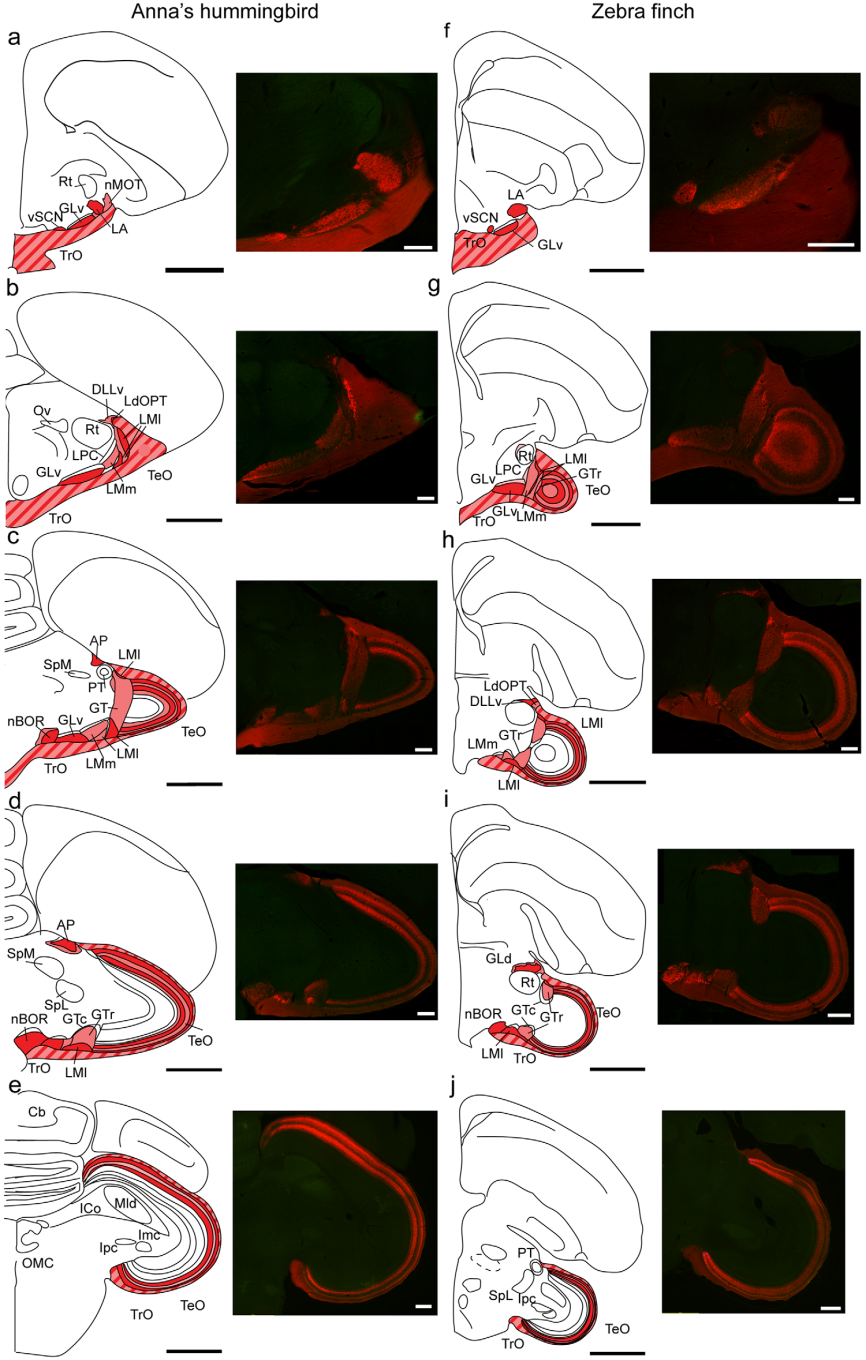
The main targets of the retinal projections in the brain of the zebra finch and Anna's hummingbird. (a–e). Each panel shows a schematic of the CTB‐labeled terminals in coronal sections through the midbrain of a zebra finch brain, presented anterior to posterior. (f–j) show the same, but for the Anna's hummingbird. Next to each schematic, there is a microphotograph of the corresponding section showing the CTB‐labeled terminals. The different shades of red in the schematic represent the intensity of the terminals. Striped areas correspond to the optic tract. In both species, similar retinorecipient areas were observed. This includes the ventral suprachiasmatic nucleus (vSCN), the thalamic ventrolateral geniculate complex, which includes the lateralis anterior thalami (LA), the ventral geniculate nucleus (GLv), and the *n. marginalis tractus optici* (nMOT). The dorsolateral geniculate complex (GLd), the pretectum (LMm, LMl, GT, AP, APd), the accessory optic system (nBOR), and the optic tectum (TeO). Also visible is the centrifugal isthmo‐optic tract (TIO). For abbreviations, see the list. Scale bars: 1 mm for schematics, 200 µm for microphotographs.

**FIGURE 4:**
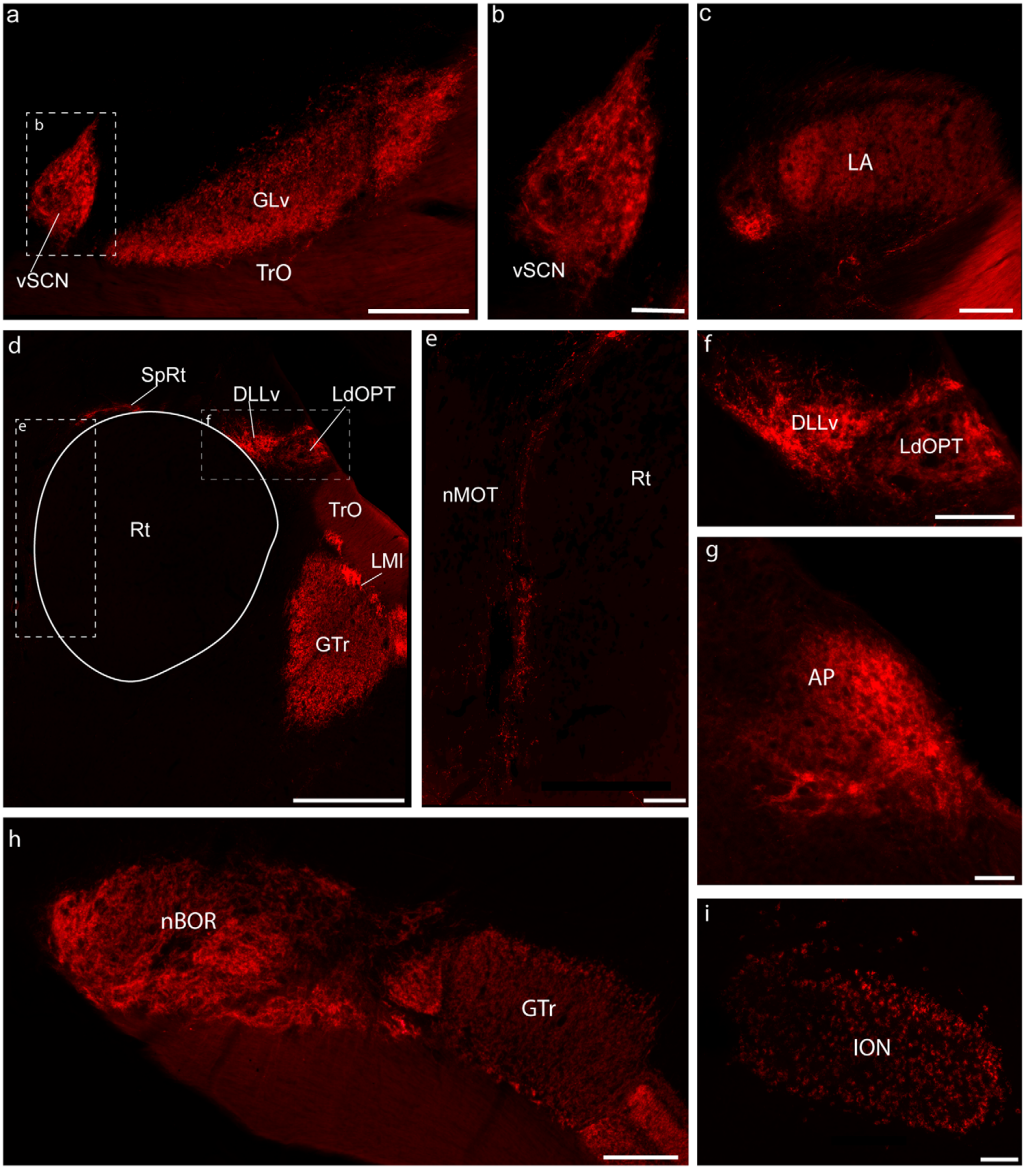
Representative photomicrographs of the main contralateral retinorecipient areas in the zebra finch. All pictures are from coronal sections. (a) Terminals in the ventral geniculate nucleus (GLv) and the visual suprachiasmatic nucleus (vSCN). (b) A higher magnification microphotograph of the inset in a, with details of the terminals in vSCN. (c) Terminals in the *lateralis anterior thalami* (LA). (d) Terminals in the ventral part of the *nucleus dorsolateralis anterior thalami, pars lateralis* (DLL), and the *nucleus lateralis dorsalis optici principalis thalami* (LdOPT), as well as in the more medially located nucleus suprarotundus (SpRt). Terminals can also be seen in the lateral part of the nucleus lentiformis mesencephali and the rostral part of the tectal grey (GTr). (e) A higher magnification microphotograph of the area in the inset in (d). Here, terminals can be seen in the medial‐most edge of the nucleus rotundus (Rt), which corresponds to the *nucleus marginalis tractus optici* (nMOT). (f) A higher magnification microphotograph of the area in the inset in (d), showing details of terminals in DLLv and LdOPT. (g) Terminals in the area pretectalis (AP). (h) Terminals in the nucleus of the basal optic root (nBOR) and the adjacent ventral part of the GTr. (i) Retrogradely labeled cells in the isthmo optic nucleus (ION). Scale bars: (a) = 200 µm; (b) and (g) = 50 µm; (e, f, and i) = 100 µm; (d, g) = 500 µm.

In both species, the injection of tracer reached the entire retina, as can be seen by the complete anteroposterior and dorsoventral labeling of retinal terminals in the TeO (Figures 2, 3, 4), which receives a full retinotopic projection from the contralateral eye (Hunt and Webster 1975). In both species, retinal projections were found in the hypothalamus, thalamus, mesencephalon, pretectum, and the accessory optic system (Figures 2, 3). Labeling was consistent across cases. As in other birds (Norren and Silver 1989; Krabichler et al. 2015; Salazar 2023), projections were largely contralateral, with only a few ipsilateral projections (see below).

### 3.2 Hypothalamus

**FIGURE 5:**
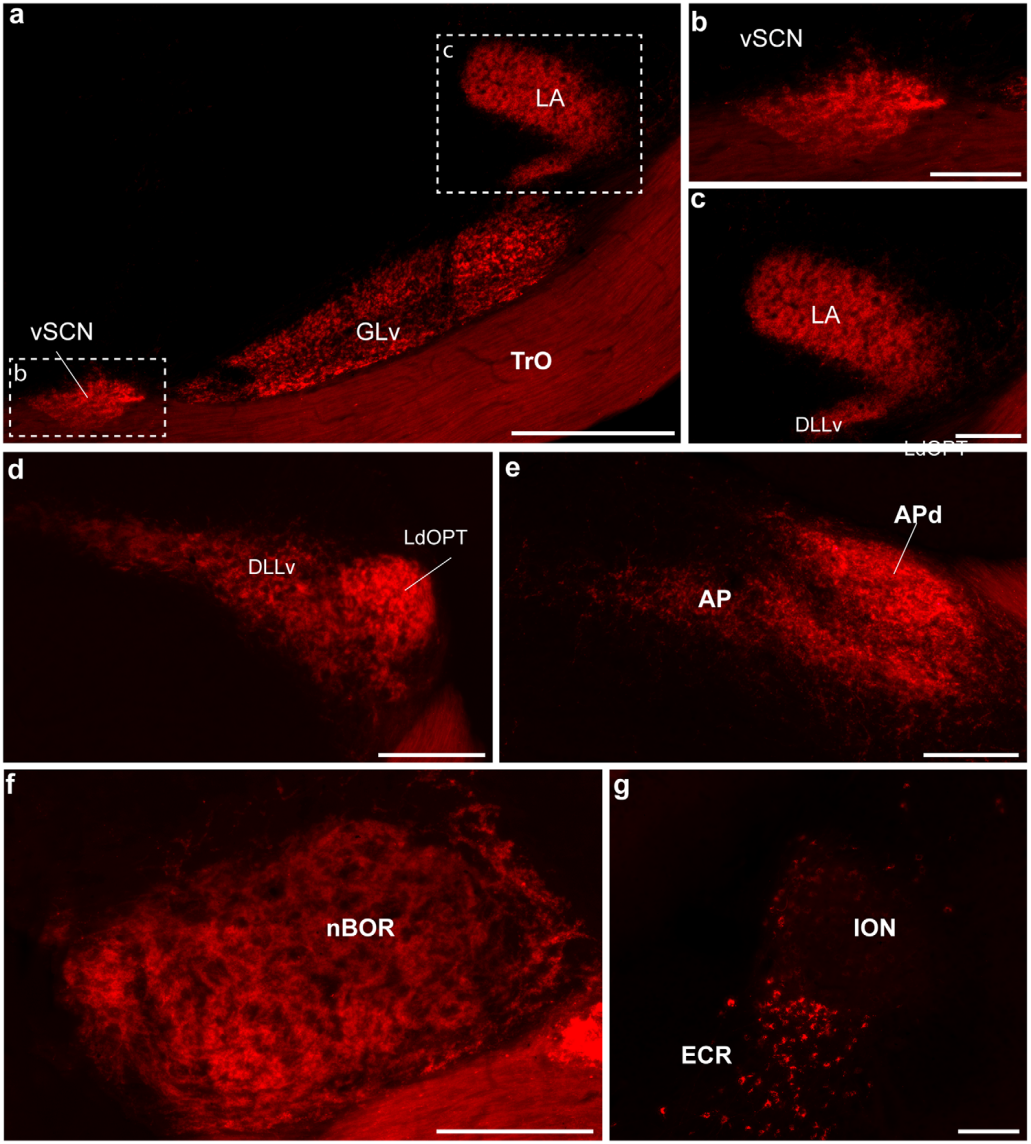
Representative photomicrographs of the main contralateral retinorecipient areas in the Anna's Hummingbird. All pictures are from coronal sections. (a) Terminal in the ventral geniculate nucleus (GLv), the visual suprachiasmatic nucleus (vSCN), and the nucleus lateralis anterior thalami (LA). (b, c) show a higher magnification microphotograph of the insets in (a), with details of the terminals in vSCN and LA, respectively. (d) Terminals in the ventral part of the nucleus dorsolateralis anterior thalami, pars lateralis (DLL), and the *nucleus lateralis dorsalis optici principalis thalami* (LdOPT). (e) Terminals in the area pretectalis (AP) and its dorsal part (APd). (f) Terminals in the nucleus of the basal optic root (nBOR) and adjacent areas. (g) Retrogradely labeled cells in the isthmo optic nucleus (ION) and the adjacent ectopic cell region. Scale bars: (a) = 300 µm, (b, g = 50 µm, (c, e, f, and i) = 100 µm, (d, g) = 500 µm.

In the ZF (Figures 3fa and 4a,b) and the AH (Figures 3a and 5a,b), the contralateral visual suprachiasmatic nucleus (vSCN) was densely labeled with retinal terminals. No terminals were observed in the medial suprachiasmatic nucleus (mSCN) of either species.

### 3.3 Thalamus

In the most anterior part of the thalamus, both in the ZF (Figures 3f and 4c) and the AH (Figures 3a and 5a,c), the contralateral anterior‐lateral nucleus (LA) was labeled densely with retina terminals. In both species, we also found low‐density terminals from the retina in the marginal nucleus of the optic tract (nMOT, Figures 4c and 5a,c), which forms an envelope around the LA (Güntürkün and Karten 1991). In the case of the ZF, but not the AH, nMOT continues caudally, medial to nucleus Rt (Figure 4d,e). In both species, the lamina interna of the GLv (GLv‐li) was densely labeled with retinal terminals throughout its anteroposterior extent (ZF: Figures 3f‐g and 4a; AH: Figures 3a‐c and 5a). In the contralateral dorsal thalamus of both species, several nuclei were labeled in the avian dorsolateral geniculate (GLd) complex (also known as the nucleus OPT). In the ZF, the principal lateral dorsal main optical nucleus of the thalamus (LdOPT), the lateral part of the dorsolateral anterior nucleus of the thalamus (DLL), and the nucleus suprarotundus (SpRt) were similarly labeled with dense retinal terminals (Figures 3g and 4d,f). In the AH, the same three nuclei in the dorsal thalamus were labeled with retinal terminals (Figure 3c), but the more lateral LdOPT was more densely labeled than DLL (Figure 5d).

### 3.4 Other Retinal Projections and the Centrifugal System

Other projections to the dorsal pretectum and accessory optic system were also similar between the two species. In both species, dense terminals were observed in the dorsal pretectum, medial to the isthmo‐optic tract, and dorsal to the PT nucleus, and in the area pretectalis (AP) and its dorsal part (APd) (ZF, Figures 3h,i and 4g; AH, Figure 5e). As expected, in both species, dense retinal projections were also found throughout the antero‐posterior extent of the nBOR, which is part of the accessory optic system (ZF, Figures 3h,i and 4h; AH, Figures 3d, 5f). Small, retrogradely labelled cells were found in the contralateral ION of the ZF (Figure 4i) as large, scattered cells in a region around and anterior to the ION in the ectopic cell region (ECR; not shown). These cells extend from the most posterior level where ION is found, near the trochlear nucleus, to the level of the oculomotor nuclei. This was also true in the AH, where small cells were labeled in the contralateral ION as well as large cells in the ECR (Figure 5g). In the ZF, but not in AH, a few ipsilaterally labeled cells could be seen in the ECR (data not shown).

### 3.5 Optic Tectum

**FIGURE 6:**
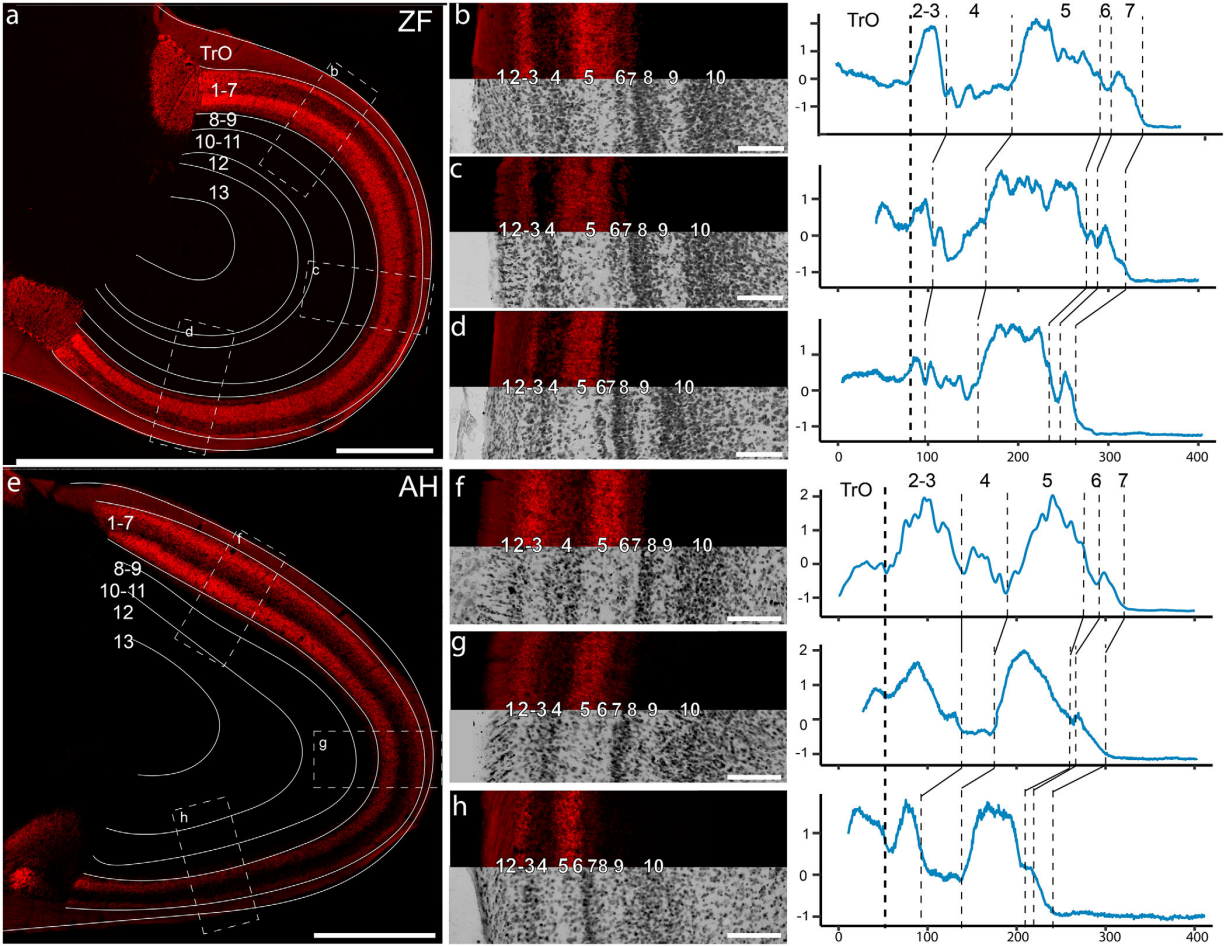
Retinal projections to the optic tectum in the zebra finch and the Anna's hummingbird. (a) Representative coronal sections through the optic tectum in the side contralateral to the eye injected with the tracer in a zebra finch (ZF). Numbers represent the optic tectum layers. (b–d) A higher magnification microphotograph of the insets in (a). Each shows the retinorecipient layers and optic track in three different areas of the optic tectum (dorsal, middle, and ventral, respectively). Next to each panel is a plot of the standardized labeling intensity of a line perpendicular to the orientation of the layers. The thick dotted line shows the border of the optic tract with layer 1, while the thinner dotted line shows the corresponding borders for each layer. (e–h) Same as above, but for the Anna's hummingbird (AH). Intensity was standardized and centered within each line. Scale bars: (a–e) = 500 µm, all others 100 µm.

**FIGURE 7:**
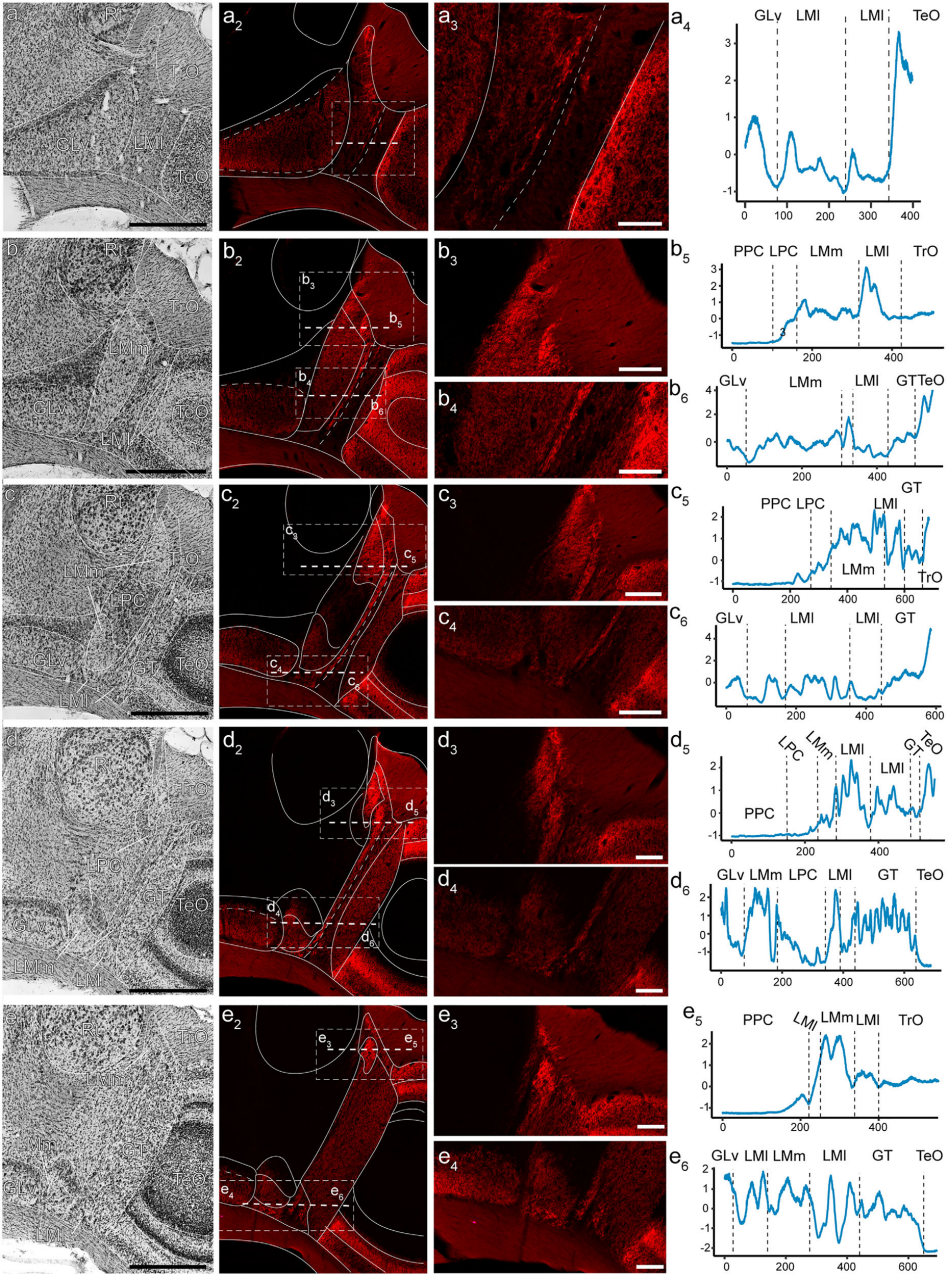
Retinal projections to the nucleus lentiformis mesencephali in the zebra finch. a_1_–e_1_ show a series organized anterior to posterior of Nissl‐stained coronal sections through the nucleus lentiformis mesencephali in the side contralateral to the eye injected with tracer. (a_2_–e_2_) A fluorescent microphotograph of the same section in a_1_–e_1_, showing the retinal terminals. Dotted lines represent the areas where the intensity of the terminal labeling was measured. Panels a_3_–e_3_ and b_4_–e_4_ show a higher magnification microphotograph of the regions where the labeling intensity was measured. Next to each of these panels (a_4_, b_5_–e_5_ and b_6_–e_6_) is the intensity labeling across the horizontal dotted line shown in the corresponding section (Panels, a–e_2_). Intensity was standardized and centered within each line. For abbreviations, see the abbreviation list. Scale bars: (a_1_–e_2_) = 500 µm, all others 100 µm.

Retinal projections were observed throughout the whole anteroposterior and dorsoventral extent of the contralateral TeO of both the ZF (Figures 2, 3, and 6a–d) and the AH (Figures 2, 3, and 6e–h). In both species, terminals were found exclusively in the external‐most layers of the TeO, Layers 1–7 (Figure 6), the *stratum griseum et fibrosum superficiale* (*SGFS*). In both species, it was clear that the density of retinal terminals was not equal across retinorecipient layers, and some layers, namely Layers 2–3 and 5, showed a much higher density of terminals than Layers 4 and 6–7. This can be seen in Figure 6, which shows a representative coronal section through the contralateral TeO of the ZF (Figure 6a–d) and AH (Figure 6e–h). For each species, three higher‐magnification panels are shown of the dorsal, mid, and ventral parts of the TeO retinorecipient layers (ZF, Figure 6b–d, AH, Figure 6f–h). In each of these areas, the intensity of the fluorescent signal was measured in a line perpendicular to the tectal layers (adjacent panels in Figure 7). Two features are apparent. First, the intensity of the retinal terminal labeling is largest and equal in Layers 2–3 and 5 across the dorsoventral extent of the TeO of both species. Similarly, intensity is the lowest in Layers 4 and 6, which correspond to layers of high neuronal density. Second, it is clear that the thickness of the tectal layers is not uniform across the dorsoventral extent of the TeO. In both species, retinorecipient layers are wider in the dorsal part of the TeO (Figure 6b,f) when compared to the ventral parts (Figure 6d,h).

### 3.6 Nucleus LM and GT

**FIGURE 8:**
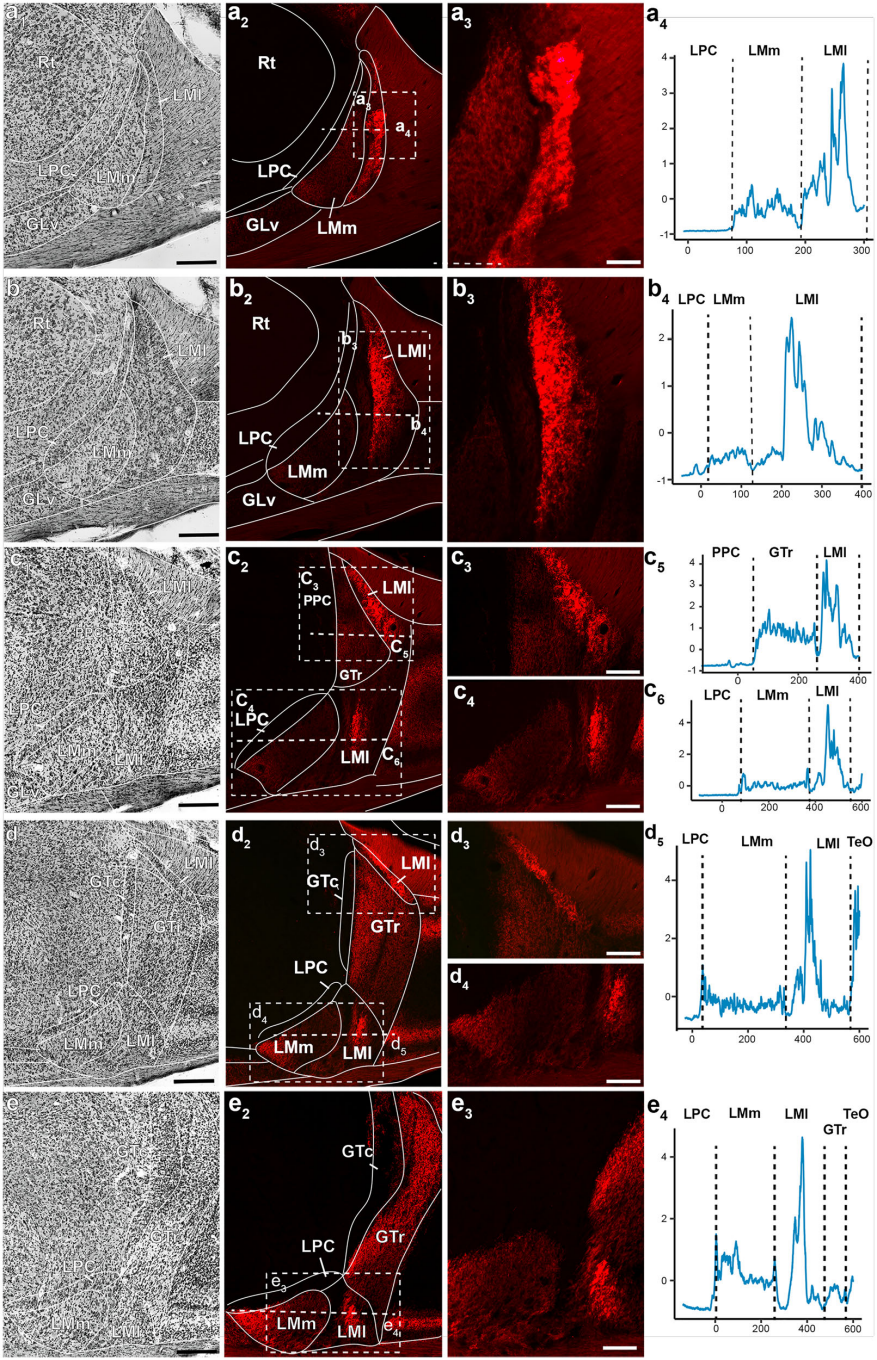
Retinal projections to the nucleus lentiformis mesencephali in the Anna's hummingbird. a_1_–e_1_ show a series from anterior to posterior of Nissl‐stained coronal sections through the nucleus lentiformis mesencephali in the side contralateral to the eye that received a tracer injection. (a_2_–e_2_) A fluorescent microphotograph of the same sections in (a_1_–e_1_), showing the retinal terminals. Dotted lines represent the areas where the intensity of the terminal labeling was measured. Panels (a_3_–e_3_) and (b_4_–e_3_) show a higher magnification microphotograph of the areas where the labeling intensity was measured. Next to each of these panels (a_4_–b_4_, c_5_–c_6_, d_5_, and e_4_) is a plot showing the intensity of labeling in the horizontal area demarcated by a dotted line in the corresponding section. Intensity was standardized and centered within each line. Scale bars: (a_1_–e_2_) = 500 µm, all other 100 µm.

Because several differences have been found between the LM of hummingbirds and other birds (see Section 1), we compared retinal terminals between the AH and the ZF in particular detail in this nucleus and the surrounding GT. Figure 7 shows five coronal sections through the LM and adjacent structures of the ZF (rostral to caudal). For each level, sections stained for Nissl are shown (Figure 7a_1_–e_1_). Adjacent panels (Figure 7a_2_–e_2_) show fluorescent images of the retinal terminals. Similar to the layers of the TeO (Figure 6), we found that terminals have varying levels of density in different parts of LM. As with the TeO (Figure 6), we measured fluorescence intensity across LM subdivisions to better quantify this. For each section shown in Figure 7, we measured fluorescence intensity in one or two lines parallel to the horizontal plane. Panels a_3_ to e_4_ of Figure 7 show a high‐magnification microphotograph of the area measured, and the adjacent panels (Figure 7a_4_–e_6_) show the measured intensity. Figure 8 shows the same for the AH, with Nissl‐stained sections (Figure 8a_1_–e_1_), adjacent fluorescent images of the same sections (Figure 8a_2_–e_2_), and areas where the intensity of terminals was measured (Figure 8a_3_–e_6_).

**FIGURE 9:**
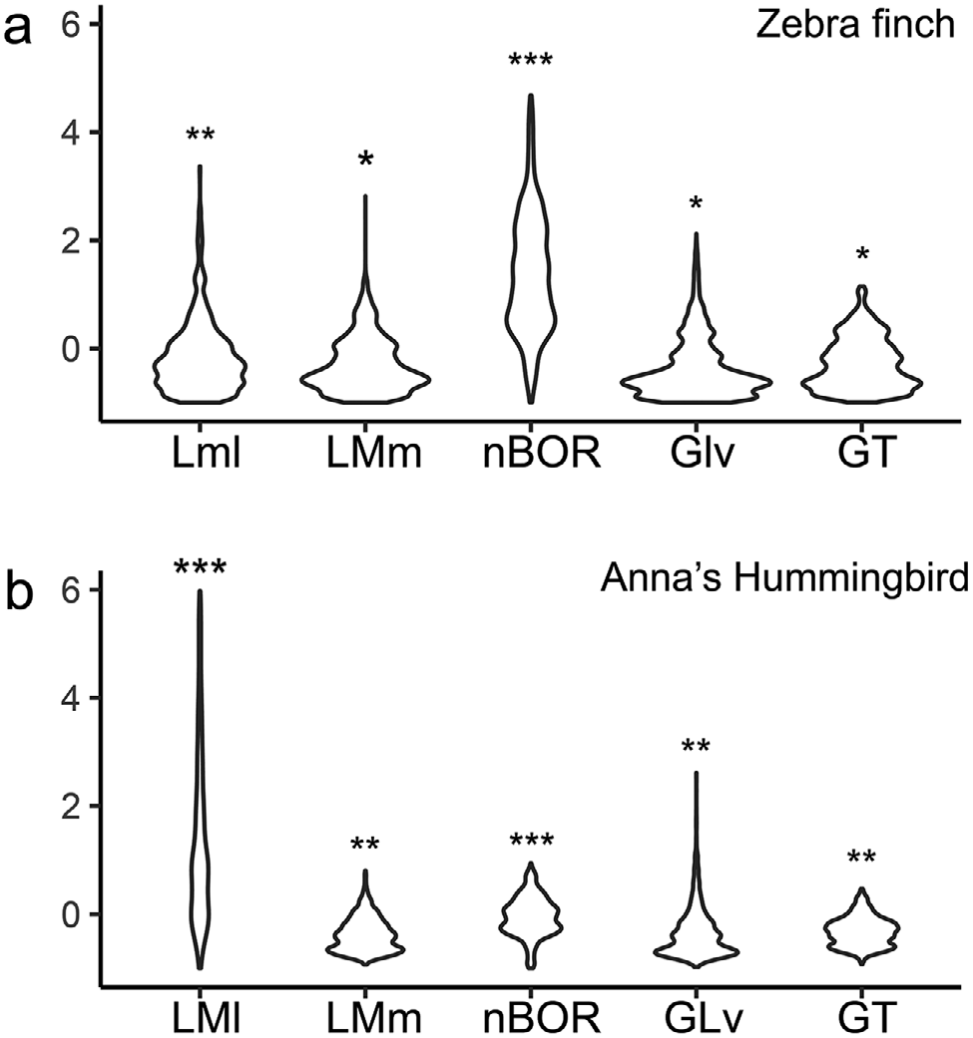
Standardized labeling intensity across retinorecipient nuclei. Violin plots of the labeling intensity after an injection in the eye of cholera toxin subunit B conjugated with a fluorescent molecule in the zebra finch (a) and the Anna's hummingbird (b) for five retinorecipient areas. Intensity values were standardized and centered within a species and a single case. Different numbers of asterisks indicate significant differences between different retinorecipient areas within each species. See the results and the abbreviation list.

In the ZF, an ANOVA shows significant differences in average intensity between LMl, LMm, Glv, GT, and nBOR (Figure 9a, ANOVA, *F*_(4, 41,336) _= 9483, *p* < 0.00001). Post hoc analysis shows that in this species, nBOR has a significantly higher average labeling intensity than the other four nuclei. Similarly, in the AH, an ANOVA also shows significant differences in average intensity between LMl, LMm, Glv, GT, and nBOR (Figure 9b; ANOVA, *F*_(4,33334)_ = 6264.8, *p* < 0.00001). A post hoc analysis shows that LMl has a significantly higher average labeling intensity than the other four nuclei.

### 3.7 Ipsilateral Projections

**FIGURE 10:**
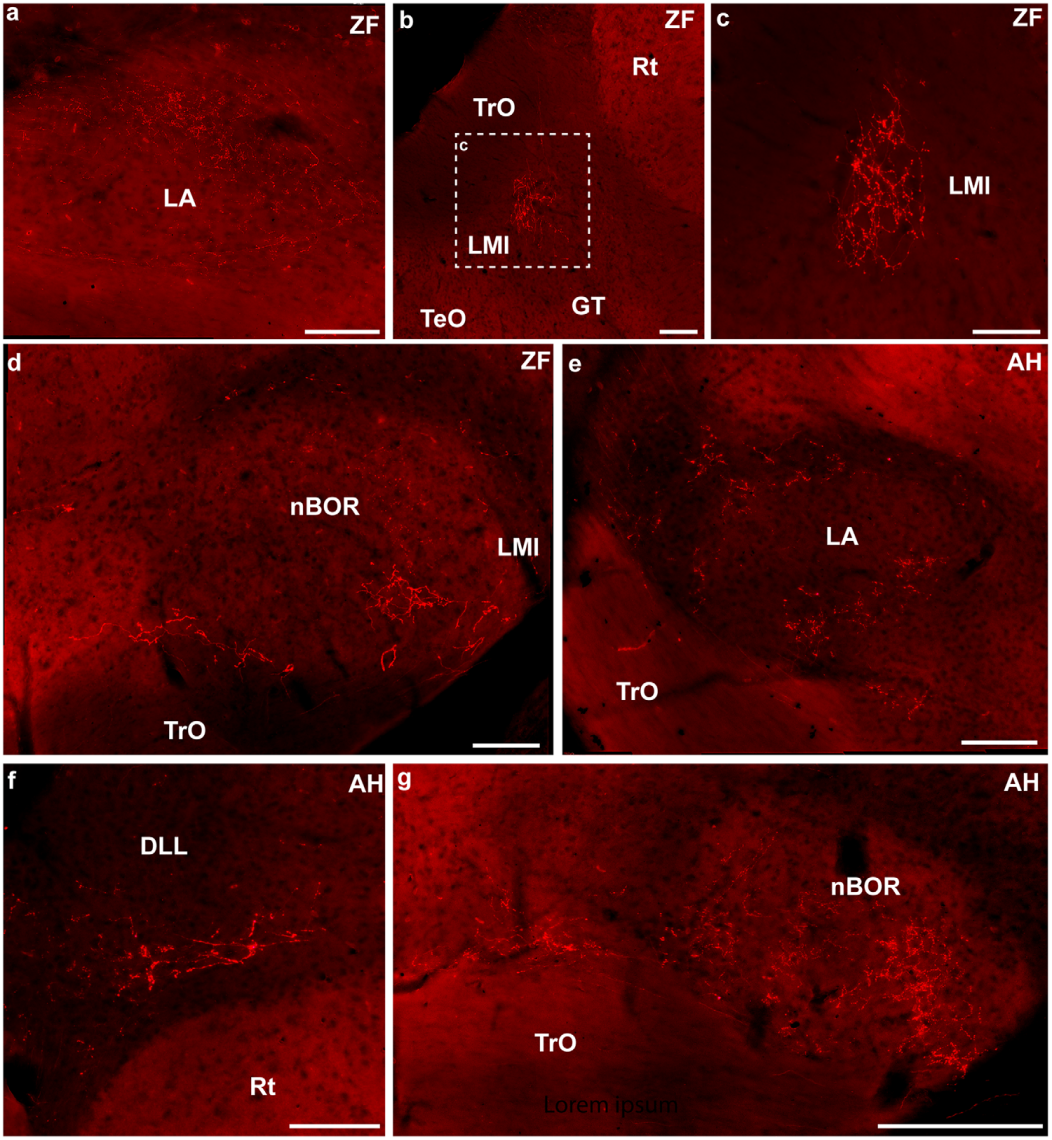
Ipsilateral projections from the retina in the zebra finch and Anna's hummingbird. All pictures are from coronal sections. (a) The nucleus lateralis anterior thalami (LA) of the zebra finch. (b) Terminals in the dorsal area of the lateral part of the ipsilateral nucleus lentiformis mesencephali (LMl), (c) A higher magnification microphotograph of the inset in (b), with details of the terminals in LMl. (d) Terminals in the ipsilateral nucleus of the basal optic root (nBOR) of the zebra finch. (e) Retinal terminals in the ipsilateral *nucleus lateralis anterior thalami* (LA) of the Anna's hummingbird. (f) Terminals in the ipsilateral *nucleus dorsolateralis anterior thalami, pars lateralis* (DLL) of the Anna's hummingbird. (g) Terminals in the ipsilateral nucleus of the basal optic root (nBOR) and the adjacent area. Scale bars: (a–f) = 100 µm; (g) = 200 µm.

A small number of labeled terminals were observed in retinorecipient areas ipsilateral to the ocular injection in both species. In the ZF, clear terminals were seen in the ipsilateral LA (Figure 10a), the lateral subdivision of the nucleus lentiformis mesencephali (LMl; Figure 10b,c), and nBOR (Figure 10d). In the AH, retinal terminals were also found in the ipsilateral LA (Figure 10e), but no terminals were found in LM, as in the ZF. In contrast, clear terminals were found in the ventral part of DLL in the ipsilateral thalamus (Figure 10f). As in the ZF, retinal terminals were also found in the ipsilateral nBOR (Figure 10g).

## 4 Discussion

In this study, we described the retinal projections of two distantly related birds, the zebra finch (*Passeriformes*) and the Anna's humingbird (*Caprimulgiformes*). Retinal targets in both birds are similar (Figures 2, 3, 4, 5, 6, 7, 8 and 10) and correspond closely to those reported in other birds from a variety of clades, including pigeons (Hunt and Webster 1975; Remy and Güntürkün 1991), *Galliformes* (Norren and Silver 1989), *Accipitiformes* (hawks and eagles, Inzunza and Bravo 1993), *tinamous* (Krabichler et al. 2015), and nightjars (Salazar 2023). This includes an almost completely decussated projection in both species, with only a few ipsilateral terminals (Figure 10), as well as a major projection to the superficial layers of the TeO. We found that in both species, projections from the retina target Layers 1–7 of TeO (Figure 6). The same has been reported in pigeons, *Galliformes*, and nocturnal and diurnal raptors (Inzunza and Bravo 1993). In contrast, in both the Chilean Tinamou (Krabichler et al. 2015) and the band‐winged nightjar (Salazar 2023), retinal projections have been reported to Layer 8 of the TeO. Given that nightjars and hummingbirds are related (Kimball et al. 2019; Chen and Field 2020; Stiller et al. 2024), the lack of projections to Layer 8 in the hummingbird suggests that this projection may be related to the particular visual ecology of nightjars, which feed on insects in the air during low light conditions at dawn and dusk (Ingels et al. 1999).

### 4.1 Optic Tectum

Our results also show that in both the ZF and AH, the thickness of tectal layers varies across the tectum such that tectal layers are thicker in the middle and dorsal parts (Figures 2 and 6). This is similar to what has been reported in other birds (Norren and Silver 1989; Karten et al. 1997; Krabichler et al. 2015). These changes in thickness and cellular density of tectal layers are likely a reflection of the topographic distribution of ganglion cells in the retina and the topographic projection to the TeO (Rodrigues et al. 2023). Areas with thicker layers correspond to areas of high density of ganglion cells in the retina. In the case of the AH, for example, Lisney et al. (2015) reported two areas of high ganglion cell density, including a central fovea, that are connected by a visual streak that runs through the middle of the retina. We also found in both species that Layer 4, although thin in the ventral tectum, still forms a distinct lamina (Figures 2 and 6) similar to what has been reported in the Chilean Tinamou (Krabichler et al. 2015). This contrasts with the pigeon, where Layer 4 is almost nonexistent in the ventral TeO (Karten et al. 1997).

Our results also show clear differences in both species in the intensity of fluorescence density of terminals in the different layers of the TeO (Figure 6). In particular, tectal layers with low cellular density (3, 5, and 7) have higher fluorescence compared to layers with a high cellular density (Layers 4 and 6, Figure 6b–d, f–h). These differences in fluorescence intensity likely reflect differences in terminal density, shape, and orientation, which in turn reflect different populations of retinal ganglion cells that project to different layers (reviewed in Luksch 2003).

### 4.2 Ipsilateral Projections

While the large majority of projections from the retina are contralateral, we found some ipsilateral projections in both species (Figure 10). This has been previously reported in the Chilean tinamou, quails, and chickens (O'Leary et al. 1983; Takatsuji et al. 1983; Weidner et al. 1985; Krabichler et al. 2015) but not in owls or hawks (Bravo and Pettigrew 1981; Inzunza and Bravo 1993). Similar to what has been reported in chickens and quails, in both hummingbirds and ZFs, LA in the thalamus and nBOR receive substantial ipsilateral projections through the anteroposterior extent. In contrast, in other targets, such as the dorsal thalamus and LM, only a few ipsilateral terminals can be seen (Figure 10). However, some differences between the two species are apparent. In the ZF, but not the hummingbird, clear terminals could be seen in the ipsilateral LM (Figure 10b, c). In contrast, we found ipsilateral projections to the dorsal thalamus (DLL) in the hummingbirds but not the ZF (Figure 10f). In the chicken, O'Leary et al. (1983) argued, based on the amount of projections that exist in the adult, that only the ipsilateral projections to nBOR are likely to be functional, while the others are only remnants of ipsilateral projections found during development. Our data support this argument. In pigeons, some nBOR cells have binocular receptive fields (Wylie and Frost 1990, 1999). While there is a substantial projection of one nBOR to the contralateral nBOR in pigeons (Brecha et al. 1980; Wylie et al. 1997), which could mediate binocular receptive fields in this nucleus, the presence of ipsilateral retinal projections to nBOR in several species of birds, including the two reported here, suggests that binocular receptive fields in nBOR could be mediated, at least in part, by direct retinal projections from both eyes.

### 4.3 Centrifugal Projections

As in other birds, in both species, injections of neural tracers in the eye not only labeled retinal terminals but also retrogradely labeled cells in the centrifugal system, that is, cells that project to the retina. In both species, cells were found contralaterally in both the ION and the surrounding ECR (Figures 4i and 5g; Woodson et al. 1995; Repérant et al. 2006; Gutierrez‐Ibanez et al. 2012; Krabichler et al. 2017). These projection cells had been previously reported in other songbirds and *Caprimulgiformes*, including swifts, which are closely related to hummingbirds (Feyerabend et al. 1994). The size and number of cells in ION vary greatly among birds (Repérant et al. 2006; Gutierrez‐Ibanez et al. 2012), and the function of these cells remains unclear. Based on Nissl‐stained material, a previous study (Gutierrez‐Ibanez et al. 2012) showed that both hummingbirds and songbirds have a well‐developed ION. Our labeling with a retrograde tracer confirms this and shows that both species also have a large number of ectopic cells. In the case of the ZF, a few cells in the ipsilateral ECR were labeled, as in other songbirds (Feyerabend et al. 1994), but no ipsilaterally labeled cells were observed in hummingbirds.

### 4.4 LM and Accessory Optic System

As with the layer of the TeO (Figure 6), the use of a fluorescent tracer allowed us to compare differences in labeling intensity in the subdivisions of LM and adjacent retinorecipient nuclei, including GT, GLv, and nBOR (Figures 7, 8, 9). In ZFs, we found that nBOR showed the highest average intensity (Figures 7 and 9a), which is significantly different from the intensity of the other retinorecipient nuclei, including both subdivisions of LM (LMm and LMl, see results). In contrast, in the hummingbird, we found that LMl shows a significantly larger average intensity of labeling compared to other retinorecipient nuclei, including nBOR (Figures 8 and 9b). As in TeO (see above), the differences in intensity likely reflect inputs from different retinal ganglion cells. In a variety of bird species, including ZFs and hummingbirds, nBOR receives projections almost exclusively from DGCs (Karten et al. 1977; Fite et al. 1981; Reiner 1981; Wylie et al. 2014; Gutierrez‐Ibanez et al. 2018). Given this, we could assume that the high labeling intensity in the nBOR of the ZF reflects the terminals from DGCs. Conversely, the higher intensity of labeling in the LMl compared to other retinorecipient nuclei of hummingbirds could reflect an increase in projections from DGCs to LMl.

This is not to say that nBOR in hummingbirds does not receive projections from DGC. We have shown in the past that this is the case (see above), and in fact, we found that in hummingbirds, nBOR has a significantly higher labeling intensity than LMm, GLv, and GT (Figure 10b, see results), consistent with the idea that projections from DGCs show higher intensity than those from other retinal ganglion cells. Alternatively, it is possible that the increased labeling intensity in the LMl of hummingbirds is due to other causes, including increased projections from other ganglion cell types or even methodological issues. Nonetheless, an increased projection from DGCs to LMl in hummingbirds could explain previous results showing anatomical and physiological differences in LM between hummingbirds and other birds. These include a hypertrophied LM in hummingbirds compared to most other birds (Iwaniuk and Wylie 2007) as well as differences in the direction preference and tuning to speed and spatial frequencies of neurons in LM between hummingbirds and other birds. (Gaede et al. 2022, 2017; Smyth et al. 2022).

### 4.5 Homologies With Mammals

It has long been held that the nucleus of the optic tract (NOT) of mammals is homologous to the LM of birds and other vertebrates. This is based on their similar connectivity, responses to optic flow, and their role, along with the accessory system, in the optokinetic response (Simpson 1984; Fite 1985; McKenna and Wallman 1985; Weber 1985; Giolli et al. 2005; Gamlin 2006). More recently, Puelles et al. (2018), based on developmental data in the chicken, have proposed different nomenclature and homologies for the two subdivisions of LM and adjacent nuclei (see Table 1). These authors proposed that LMl is homologous to the lateral terminal nucleus of mammals, while LMm, LPC, and PPC are homologous to different subdivisions of the anterior pretectal nucleus of mammals. They also propose that NOT is not part of the pretectum, as it has always been assumed (Gamlin 2006), but homologous to parts of the GT (Puelles et al. 2018; Puelles 2022). This proposal is intriguing, as it implies that the aforementioned similarities between NOT and LM are the product of convergent evolution. The proposed homology is particularly striking for LMm and parts of the anterior pretectal nucleus, as there is no evidence that the latter responds to optic flow, it is not reciprocally connected with the medial terminal nucleus, nor is it involved in image stabilization (Rees and Roberts 1993; Giber et al. 2008). While the anterior pretectal nucleus does receive retinal inputs in some species of mammals (Hutchins 1991), it is not connected to other visual areas (including the medial terminal nucleus), and in fact, it is involved in touch and pain sensation (Rees and Roberts 1993; Genaro and Prado 2021). We do acknowledge that the two subdivisions of LM in birds are likely two distinct nuclei. As such, the homologies of LMl and LMm to NOT and the lateral and dorsal subdivisions of the terminal nuclei in mammals are unclear. Ultimately, it is good to keep in mind that while establishing homologies exclusively based on hodology or function may be a fool's errand, using similar gene expressions or developmental origin cannot supply an absolute criterion for determining homology either (Beer 1974; Hall 2003; Faunes et al. 2015).

## Author Contributions

Study concept and design: Douglas R. Wylie, Douglas L. Altshuler, Andrea H. Gaede, Cristián Gutiérrez‐Ibáñez. Performed experiments and processed tissue: Andrea H. Gaede, Cristián Gutiérrez‐Ibáñez. Microscopy and Image Acquisition: Julia A. Bowen, Cristián Gutiérrez‐Ibáñez. Analysis: Douglas R. Wylie, Cristián Gutiérrez‐Ibáñez, Julia A. Bowen. Drafting of the article: Douglas R. Wylie, Cristián Gutiérrez‐Ibáñez. Construction of figures: Julia A. Bowen, Cristián Gutiérrez‐Ibáñez. Critical revision of the article for important intellectual content: Douglas L. Altshuler, Andrea H. Gaede. Obtained funding: Douglas L. Altshuler, Douglas R. Wylie. Student supervision: Douglas L. Altshuler, Douglas R. Wylie, Cristián Gutiérrez‐Ibáñez. All authors had full access to all the data in the study and take responsibility for the integrity of the data and the accuracy of the data analysis.

## Conflicts of Interest

The authors declare no conflict of interest.

## Peer Review

The peer review history for this article is available at https://publons.com/publon/10.1002/cne.70087.

## Data Availability

The data that support the findings of this study are available on request from the corresponding author, C.G.I.

## References

[cne70087-bib-0001] Altshuler, D. L. , and R. Dudley . 2002. “The Ecological and Evolutionary Interface of Hummingbird Flight Physiology.” Journal of Experimental Biology 205: 2325–2336. 12124359 10.1242/jeb.205.16.2325

[cne70087-bib-0002] Baliga, V. B. , R. Dakin , D. R. Wylie , and D. L. Altshuler . 2024. “Hummingbirds Use Distinct Control Strategies for Forward and Hovering Flight.” Proceedings of the Royal Society B: Biological Sciences 291: 20232155. 10.1098/rspb.2023.2155 PMC10777153 38196357

[cne70087-bib-0003] Beer, G. D 1974. Homology, an Unsolved Problem. Carolina Biological Supply Company.

[cne70087-bib-0004] Bellintani‐Guardia, B. , and M. Ott . 2002. “Displaced Retinal Ganglion Cells Project to the Accessory Optic System in the Chameleon (*Chamaeleo calyptratus*).” Experimental Brain Research 145: 56–63. 12070745 10.1007/s00221-002-1091-z

[cne70087-bib-0005] Benowitz, L. I. , and H. J. Karten . 1976. “Organization of the Tectofugal Visual Pathway in the Pigeon: A Retrograde Transport Study.” Journal of Comparative Neurology 167: 503–520. 1270632 10.1002/cne.901670407

[cne70087-bib-0006] Bravo, H. , and J. D. Pettigrew . 1981. “The Distribution of Neurons Projecting From the Retina and Visual Cortex to the Thalamus and Tectum Opticum of the Barn Owl, *Tyto alba*, and the Burrowing Owl, Speotyto Cunicularia.” Journal of Comparative Neurology 199: 419–441. 7263955 10.1002/cne.901990307

[cne70087-bib-0007] Brecha, N. , H. J. Karten , and S. P. Hunt . 1980. “Projections of the Nucleus of the Basal Optic Root in the Pigeon: An Autoradiographic and Horseradish Peroxidase Study.” Journal of Comparative Neurology 189: 615–670. 7381044 10.1002/cne.901890404

[cne70087-bib-0008] Chen, A. , and D. Field . 2020. “Phylogenetic Definitions for *Caprimulgimorphae* (*Aves*) and Major Constituent Clades Under the International Code of Phylogenetic Nomenclature.” Vertebrate Zoology 70: 571–585.

[cne70087-bib-0009] Dakin, R. , T. K. Fellows , and D. L. Altshuler . 2016. “Visual Guidance of Forward Flight in Hummingbirds Reveals Control Based on Image Features Instead of Pattern Velocity.” Proceedings of the National Academy of Sciences of the United States of America 113: 8849–8854. 27432982 10.1073/pnas.1603221113 PMC4978297

[cne70087-bib-0010] Dhande, O. S. , M. E. Estevez , L. E. Quattrochi , et al. 2013. “Genetic Dissection of Retinal Inputs to Brainstem Nuclei Controlling Image Stabilization.” Journal of Neuroscience 33: 17797–17813. 24198370 10.1523/JNEUROSCI.2778-13.2013 PMC3818553

[cne70087-bib-0011] Ebbesson, S. O. E 1970. “On the Organization of Central Visual Pathways in Vertebrates.” Brain Behavior and Evolution 3: 178–194. 5001240 10.1159/000125470

[cne70087-bib-0012] Faunes, M. , J. Francisco Botelho , P. Ahumada Galleguillos , and J. Mpodozis . 2015. “On the Hodological Criterion for Homology.” Frontiers in Neuroscience 9: 223. 10.3389/fnins.2015.00223. 26157357 PMC4477164

[cne70087-bib-0013] Feyerabend, B. , C. R. Malz , and D. L. Meyer . 1994. “Birds That Feed‐on‐the‐Wing Have Few Isthmo‐Optic Neurons.” Neuroscience Letters 182: 66–68. 7891890 10.1016/0304-3940(94)90207-0

[cne70087-bib-0014] Fite, K. V 1985. “Pretectal and Accessory‐Optic Visual Nuclei of Fish, Amphibia and Reptiles: Theme and Variations.” Brain Behavior and Evolution 26: 81–90. 10.1159/000118769 3907745

[cne70087-bib-0015] Fite, K. V. , N. Brecha , H. J. Karten , and S. P. Hunt . 1981. “Displaced Ganglion Cells and the Accessory Optic System of Pigeon.” Journal of Comparative Neurology 195: 279–288. 7251927 10.1002/cne.901950208

[cne70087-bib-0016] Fredes, F. , S. Tapia , J. C. Letelier , G. Marín , and J. Mpodozis . 2010. “Topographic Arrangement of the Rotundo‐Entopallial Projection in the Pigeon (*Columba livia*).” Journal of Comparative Neurology 518: 4342–4361. 20853511 10.1002/cne.22460

[cne70087-bib-0017] Gaede, A. H. , V. B. Baliga , G. Smyth , C. Gutiérrez‐Ibáñez , D. L. Altshuler , and D. R. Wylie . 2022. “Response Properties of Optic Flow Neurons in the Accessory Optic System of Hummingbirds Versus Zebra Finches and Pigeons.” Journal of Neurophysiology 127: 130–144. 34851761 10.1152/jn.00437.2021

[cne70087-bib-0018] Gaede, A. H. , B. Goller , J. P. M. Lam , D. R. Wylie , and D. L. Altshuler . 2017. “Neurons Responsive to Global Visual Motion Have Unique Tuning Properties in Hummingbirds.” Current Biology 27: 279–285. 28065606 10.1016/j.cub.2016.11.041

[cne70087-bib-0088] Gaede, A. H. , C. Gutierrez‐Ibanez , M. S. Armstrong , D. L. Altshuler , and D. R. Wylie . 2019. “Pretectal projections to the oculomotor cerebellum in hummingbirds (Calypte anna), zebra finches (Taeniopygia guttata), and pigeons (Columba livia).” Journal of Comparative Neurology 527, no. 16: 2644–2658. 30950058 10.1002/cne.24697

[cne70087-bib-0089] Gaede, A. H. , C. Gutiérrez‐Ibáñez , P. H. Wu , M. C. Pilon , D. L. Altshuler , and D. R. Wylie . 2024. “Topography of visual and somatosensory inputs to the pontine nuclei in zebra finches (Taeniopygia guttata).” Journal of Comparative Neurology 532: e25556. 37938923 10.1002/cne.25556

[cne70087-bib-0019] Gamlin, P. D. R 2006. “The Pretectum: Connections and Oculomotor‐Related Roles.” Progress in Brain Research 151: 379–405. 16221595 10.1016/S0079-6123(05)51012-4

[cne70087-bib-0020] Gamlin, P. D. R. , and D. H. Cohen . 1988. “Retinal Projections to the Pretectum in the Pigeon (*Columba livia*).” Journal of Comparative Neurology 269: 1–17. 3360999 10.1002/cne.902690102

[cne70087-bib-0021] Genaro, K. , and W. A. Prado . 2021. “The Role of the Anterior Pretectal Nucleus in Pain Modulation: A Comprehensive Review.” European Journal of Neuroscience 54: 4358–4380. 10.1111/ejn.15255 33909941

[cne70087-bib-0022] Giber, K. , A. Slézia , H. Bokor , et al. 2008. “Heterogeneous Output Pathways Link the Anterior Pretectal Nucleus With the *Zona incerta* and the Thalamus in Rat.” Journal of Comparative Neurology 506: 122–140. 17990275 10.1002/cne.21545 PMC2670449

[cne70087-bib-0023] Gibson, J. J 1954. “The Visual Perception of Objective Motion and Subjective Movement.” Psychological Review 61: 304–314. 13204493 10.1037/h0061885

[cne70087-bib-0024] Giolli, R. A. , R. H. I. Blanks , and F. Lui . 2005. “The Accessory Optic System: Basic Organization With an Update on Connectivity, Neurochemistry, and Function.” Progress in Brain Research 151: 407–440. 10.1016/S0079-6123(05)51013-6 16221596

[cne70087-bib-0025] Güntürkün, O. , and H. J. Karten . 1991. “An Immunocytochemical Analysis of the Lateral Geniculate Complex in the Pigeon (*Columba livia*).” Journal of Comparative Neurology 314: 721–749. 1687743 10.1002/cne.903140407

[cne70087-bib-0026] Gutierrez‐Ibanez, C. , A. H. Gaede , D. MaxR , D. L. Altshuler , and D. R. Wylie . 2018. “The Retinal Projection to the Nucleus Lentiformis Mesencephali in Zebra Finch (*Taeniopygia guttata*) and Anna's Hummingbird (*Calypte anna*).” Journal of Comparative Physiology A 204: 369–376. 10.1007/s00359-018-1245-5 29340763

[cne70087-bib-0027] Gutierrez‐Ibanez, C. , A. N. Iwaniuk , T. Lisney , M. Faunes , G. J. Marín , and D. R. Wylie . 2012. “Functional Implications of Species Differences in the Size and Morphology of the Isthmo Optic Nucleus (ION) in Birds.” PLoS ONE 7: e37816. 22666395 10.1371/journal.pone.0037816 PMC3362605

[cne70087-bib-0028] Gutiérrez‐Ibáñez, C. , D. R. Wylie , and D. L. Altshuler . 2023. “From the Eye to the Wing: Neural Circuits for Transforming Optic Flow Into Motor Output in Avian Flight.” Journal of Comparative Physiology A 209: 839–854. 10.1007/s00359-023-01663-5 37542566

[cne70087-bib-0029] Hall, B. K 2003. “Descent With Modification: the Unity Underlying Homology and Homoplasy as Seen Through an Analysis of Development and Evolution.” Biological Reviews 78: 409–433. 14558591 10.1017/s1464793102006097

[cne70087-bib-0030] Hodos, W. , and A. B. Butler . 2008. “Evolution of Sensory Pathways in Vertebrates.” Brain Behavior and Evolution 50: 189–197. 10.1159/000113333 9310194

[cne70087-bib-0031] Hoffmann, K.‐P. , and A. Schoppmann . 1981. “A Quantitative Analysis of the Direction‐Specific Response of Neurons in the Cat's Nucleus of the Optic Tract.” Experimental Brain Research 42: 146–157. 7262211 10.1007/BF00236901

[cne70087-bib-0032] Horowitz, S. S. , C. A. Cheney , and J. A. Simmons . 2004. “Interaction of Vestibular, Echolocation, and Visual Modalities Guiding Flight by the Big Brown Bat, *Eptesicus fuscus* .” Journal of Vestibular Research 14: 17–32. 15156093

[cne70087-bib-0033] Hunt, S. P. , and K. E. Webster . 1975. “The Projection of the Retina Upon the Optic Tectum of the Pigeon.” Journal of Comparative Neurology 162: 433–445. 1150928 10.1002/cne.901620403

[cne70087-bib-0034] Hutchins, B 1991. “Evidence for a Direct Retinal Projection to the Anterior Pretectal Nucleus in the Cat.” Brain Research 561: 169–173. 1797344 10.1016/0006-8993(91)90764-m

[cne70087-bib-0035] Ibbotson, M. R. , and N. S. C. Price . 2001. “Spatiotemporal Tuning of Directional Neurons in Mammalian and Avian Pretectum: A Comparison of Physiological Properties.” Journal of Neurophysiology 86: 2621–2624. 11698548 10.1152/jn.2001.86.5.2621

[cne70087-bib-0036] Ingels, J. , Y. Oniki , and E. O. Willis . 1999. “Opportunistic Adaptations to Man‐Induced Habitat Changes by some South American Caprimulgidae.” Revista Brasileira De Biologia 59: 563–566. 23505644 10.1590/s0034-71081999000400005

[cne70087-bib-0037] Inzunza, O. , and H. Bravo . 1993. “Foveal Topography in the Optic Nerve and Primary Visual Centers in Falconiforms.” Anatomical Record 235: 622–631. 8465993 10.1002/ar.1092350415

[cne70087-bib-0038] Iwaniuk, A. N. , and D. R. W. Wylie . 2007. “Neural Specialization for Hovering in Hummingbirds: Hypertrophy of the Pretectal Nucleus Lentiformis Mesencephali.” Journal of Comparative Neurology 500: 211–221. 17111358 10.1002/cne.21098

[cne70087-bib-0039] Karten, H. J. , K. Cox , and J. Mpodozis . 1997. “Two Distinct Populations of Tectal Neurons Have Unique Connections Within the Retinotectorotundal Pathway of the Pigeon (*Columba livia*).” Journal of Comparative Neurology 387: 449–465. 9335427

[cne70087-bib-0040] Karten, H. J. , and W. Hodos . 1970. “Telencephalic Projections of the Nucleus Rotundus in the Pigeon (*Columba livia*).” Journal of Comparative Neurology 140: 35–51. 5459211 10.1002/cne.901400103

[cne70087-bib-0041] Karten, H. J. , W. Hodos , W. J. H. Nauta , and A. M. Revzin . 1973. “Neural Connections of the “Visual Wulst” of the Avian Telencephalon. Experimental Studies in the Pigeon (*Columba livia*) and Owl (*Speotyto cunicularia*).” Journal of Comparative Neurology 150: 253–277. 4721779 10.1002/cne.901500303

[cne70087-bib-0091] Karten, H. J. , W. Hodos , and W. J. Nauta . 1967. A stereotaxic atlas of the brain of the pigeon: (Columba Livia). Baltimore: Johns Hopkins Press.

[cne70087-bib-0042] Karten, J. H. , K. V. Fite , and N. Brecha . 1977. “Specific Projection of Displaced Retinal Ganglion Cells Upon the Accessory Optic System in the Pigeon (*Columbia livia*).” Proceedings of the National Academy of Sciences of the United States of America 74: 1753–1756. 266216 10.1073/pnas.74.4.1753 PMC430872

[cne70087-bib-0043] Kimball, R. T. , C. H. Oliveros , N. Wang , et al. 2019. “A Phylogenomic Supertree of Birds.” Diversity 11: 109.

[cne70087-bib-0044] Krabichler, Q. , T. Vega‐Zuniga , D. Carrasco , et al. 2017. “The Centrifugal Visual System of a Palaeognathous Bird, the Chilean Tinamou (*Nothoprocta perdicaria*).” Journal of Comparative Neurology 525: 2514–2534. 28256705 10.1002/cne.24195

[cne70087-bib-0045] Krabichler, Q. , T. Vega‐Zuniga , C. Morales , H. Luksch , and G. J. Marín . 2015. “The Visual System of a Palaeognathous Bird: Visual Field, Retinal Topography and Retino‐Central Connections in the Chilean Tinamou (*Nothoprocta perdicaria*).” Journal of Comparative Neurology 523: 226–250. 25224833 10.1002/cne.23676

[cne70087-bib-0046] Krützfeldt, N. O. E. , and J. M. Wild . 2004. “Definition and Connections of the Entopallium in the Zebra Finch (*Taeniopygia guttata*).” Journal of Comparative Neurology 468: 452–465. 14681937 10.1002/cne.10972

[cne70087-bib-0047] Lisney, T. J. , D. R. Wylie , J. Kolominsky , and A. N. Iwaniuk . 2015. “Eye Morphology and Retinal Topography in Hummingbirds (*Trochilidae*: *Aves*).” Brain, Behavior and Evolution 86: 176–190. 26587582 10.1159/000441834

[cne70087-bib-0048] Luksch, H 2003. “Cytoarchitecture of the Avian Optic Tectum: Neuronal Substrate for Cellular Computation.” Reviews in the Neurosciences 14: 85–106. 12929921 10.1515/revneuro.2003.14.1-2.85

[cne70087-bib-0049] McKenna, O. C. , and J. Wallman . 1985. “Accessory Optic System and Pretectum of Birds: Comparisons With Those of Other Vertebrates.” Brain, Behavior and Evolution 26: 91–116. 3907746 10.1159/000118770

[cne70087-bib-0050] Miceli, D. , J. Peyrichoux , and J. Repe´rant . 1975. “The Retino‐Thalamo‐Hyperstriatal Pathway in the Pigeon (*Columba livia*).” Brain Research 100: 125–131. 1182505 10.1016/0006-8993(75)90247-4

[cne70087-bib-0051] Mpodozis, J. , J.‐C. Letelier , M. L. Concha , and H. Maturana . 1995. “Conduction Velocity Groups in the Retino‐Tectal and Retino‐Thalamic Visual Pathways of the Pigeon (*Columba livia*).” International Journal of Neuroscience 81: 123–136. 7775067 10.3109/00207459509015304

[cne70087-bib-0052] Mustari, M. J. , and A. F. Fuchs . 1990. “Discharge Patterns of Neurons in the Pretectal Nucleus of the Optic Tract (NOT) in the Behaving Primate.” Journal of Neurophysiology 64: 77–90. 2388076 10.1152/jn.1990.64.1.77

[cne70087-bib-0053] Nakayama, K 1981. “Differential Motion Hyperacuity Under Conditions of Common Image Motion.” Vision Research 21: 1475–1482. 7331244 10.1016/0042-6989(81)90218-2

[cne70087-bib-0054] Norgren Jr R. B. , and R. Silver . 1989. “Retinohypothalamic Projections and the Suprachiasmatic Nucleus in Birds.” Brain Behavior and Evolution 34: 73–83. 2819412 10.1159/000116493

[cne70087-bib-0055] Norren, R. B. , and R. Silver . 1989. “Retinal Projections in Quail (*Coturnix coturnix*).” Visual Neuroscience 3: 377–387. 2487114 10.1017/s095252380000554x

[cne70087-bib-0056] O'Leary, D. D. M. , C. R. Gerfen , and W. M. Cowan . 1983. “The Development and Restriction of the Ipsilateral Retinofugal Projection in the Chick.” Developmental Brain Research 10: 93–109. 10.1016/0165-3806(83)90124-4 6652510

[cne70087-bib-0057] Puelles, L 2022. “Prosomeric Classification of Retinorecipient Centers: A New Causal Scenario.” Brain Structure and Function 227: 1171–1193. 35171343 10.1007/s00429-022-02461-6

[cne70087-bib-0058] Puelles, L. , M. Martinez‐de‐la‐Torre , S. Martinez , C. Watson , and G. Paxinos . 2018. The Chick Brain in Stereotaxic Coordinates and Alternate Stains: Featuring Neuromeric Divisions and Mammalian Homologies. Academic Press.

[cne70087-bib-0059] R Core Team . 2022. “R: A Language and Environment for Statistical Computing.” https://www.R‐project.org/.

[cne70087-bib-0060] Rees, H. , and M. H. T. Roberts . 1993. “The Anterior Pretectal Nucleus: A Proposed Role in Sensory Processing.” Pain 53: 121. 8336983 10.1016/0304-3959(93)90072-W

[cne70087-bib-0061] Reiner, A 1981. “A Projection of Displaced Ganglion Cells and Giant Ganglion Cells to the Accessory Optic Nuclei in Turtle.” Brain Research 204: 403–409. 7459635 10.1016/0006-8993(81)90598-9

[cne70087-bib-0062] Reiner, A. , and H. J. Karten . 1982. “Laminar Distribution of the Cells of Origin of the Descending Tectofugal Pathways in the Pigeon (*Columba livia*).” Journal of Comparative Neurology 204: 165–187. 7056890 10.1002/cne.902040206

[cne70087-bib-0063] Remy, M. , and O. Güntürkün . 1991. “Retinal Afferents to the Tectum Opticum and the Nucleus Opticus Principalis Thalami in the Pigeon.” Journal of Comparative Neurology 305: 57–70. 1709649 10.1002/cne.903050107

[cne70087-bib-0064] Repérant, J. , R. Ward , D. Miceli , et al. 2006. “The Centrifugal Visual System of Vertebrates: a Comparative Analysis of Its Functional Anatomical Organization.” Brain Research Reviews 52: 1–57. 16469387 10.1016/j.brainresrev.2005.11.008

[cne70087-bib-0065] Riss, W. , and J. S. Jakway . 1970. “A Perspective on the Fundamental Retinal Projections of Vertebrates.” Brain Behavior and Evolution 3: 30–35. 5522351 10.1159/000125461

[cne70087-bib-0066] Rodieck, R. , R. Brening , and M. Watanabe . 1993. “The Origin of Parallel Visual Pathways.” In Proceedings of the Retinal Research Foundation Symposium , 117–144. MIT Press.

[cne70087-bib-0067] Rodrigues, T. , L. Dib , É. Bréthaut , M. M. Matter , L. Matter‐Sadzinski , and J.‐M. Matter . 2023. “Increased Neuron Density in the Midbrain of a Foveate Bird, Pigeon, Results From Profound Change in Tissue Morphogenesis.” Developmental Biology 502: 77–98. 37400051 10.1016/j.ydbio.2023.06.021

[cne70087-bib-0068] Ros, I. G. , and A. A. Biewener . 2016. “Optic Flow Stabilizes Flight in Ruby‐Throated Hummingbirds.” Journal of Experimental Biology 219, no. 16: 2443–2448. 10.1242/jeb.128488. 27284072

[cne70087-bib-0069] Salazar, J. E 2023. “Descripción neuroetológica de los sistemas sensoriales asociados al forrajeo en un ave insectívora nocturna, la Gallina ciega (*Systellura longirostris*).” Master's Thesis, Universidad de Chile. https://repositorio.uchile.cl/handle/2250/193302.

[cne70087-bib-0070] Shimizu, T. , K. Cox , H. J. Karten , and L. R. G. Britto . 1994. “Cholera Toxin Mapping of Retinal Projections in Pigeons (*Columba livia*), With Emphasis on Retinohypothalamic Connections.” Visual Neuroscience 11: 441–446. 8038120 10.1017/s0952523800002376

[cne70087-bib-0071] Simpson, J. I 1984. “The Accessory Optic System.” Annual Review of Neuroscience 7: 13–41. 10.1146/annurev.ne.07.030184.000305 6370078

[cne70087-bib-0072] Smyth, G. , V. B. Baliga , A. H. Gaede , D. R. Wylie , and D. L. Altshuler . 2022. “Specializations in Optic Flow Encoding in the Pretectum of Hummingbirds and Zebra Finches.” Current Biology 32: 2772–2779. 35609607 10.1016/j.cub.2022.04.076

[cne70087-bib-0073] Stiller, J. , S. Feng , A.‐A. Chowdhury , et al. 2024. “Complexity of Avian Evolution Revealed by Family‐Level Genomes.” Nature 629: 851–860. 38560995 10.1038/s41586-024-07323-1 PMC11111414

[cne70087-bib-0074] Takatsuji, K. , H. Ito , and H. Masai . 1983. “Ipsilateral Retinal Projections in Japanese Quail, *Coturnix Coturnix japonica* .” Brain Research Bulletin 10: 53–56. 6824967 10.1016/0361-9230(83)90074-6

[cne70087-bib-0075] Waespe, W. , and V. Henn . 1987. “Gaze Stabilization in the primate.” In Reviews of Physiology, Biochemistry and Pharmacology. Vol. 106, 37–125. Springer Berlin Heidelberg. 10.1007/BFb0027575. 3303269

[cne70087-bib-0076] Weber, J. T 1985. “Pretectal Complex and Accessory Optic System of Primates.” Brain Behavior and Evolution 26: 117–128. 3907744 10.1159/000118771

[cne70087-bib-0077] Weidner, C. , J. Repérant , D. Miceli , M. Haby , and J. P. Rio . 1985. “An Anatomical Study of Ipsilateral Retinal Projections in the Quail Using Radioautographic, Horseradish Peroxidase, Fluorescence and Degeneration Techniques.” Brain Research 340: 99–108. 4027648 10.1016/0006-8993(85)90778-4

[cne70087-bib-0078] Westheimer, G. , and S. P. McKee . 1975. “Visual Acuity in the Presence of Retinal‐Image Motion.” Journal of the Optical Society of America 65: 847. 1142031 10.1364/josa.65.000847

[cne70087-bib-0079] Wilson, V. J. , and G. M. Jones . 1979. “The Vestibuloocular System.” In Mammalian Vestibular Physiology, 249–318. Springer. https://link.springer.com/chapter/10.1007/978‐1‐4757‐5702‐6_8.

[cne70087-bib-0080] Winterson, B. J. , and S. E. Brauth . 1985. “Direction‐Selective Single Units in the Nucleus Lentiformis Mesencephali of the Pigeon (*Columba livia*).” Experimental Brain Research 60: 215–226. 4054266 10.1007/BF00235916

[cne70087-bib-0081] Woodson, W. , T. Shimizu , J. M. Wild , J. Schimke , K. Cox , and H. J. Karten . 1995. “Centrifugal Projections Upon the Retina: an Anterograde Tracing Study in the Pigeon (*Columba livia*).” Journal of Comparative Neurology 362: 489–509. 8636463 10.1002/cne.903620405

[cne70087-bib-0082] Wylie, D. R. , and B. J. Frost . 1990. “Binocular Neurons in the Nucleus of the Basal Optic Root (nBOR) of the Pigeon Are Selective for Either Translational or Rotational Visual Flow.” Visual Neuroscience 5: 489. 2288897 10.1017/s0952523800000614

[cne70087-bib-0083] Wylie, D. R. , J. Kolominsky , D. J. Graham , T. J. Lisney , and C. Gutierrez‐Ibanez . 2014. “Retinal Projection to the Pretectal Nucleus Lentiformis Mesencephali in Pigeons (*Columba livia*).” Journal of Comparative Neurology 522: 3928–3942. 25044056 10.1002/cne.23649

[cne70087-bib-0084] Wylie, D. R. , B. Linkenhoker , and K. L. Lau . 1997. “Projections of the Nucleus of the Basal Optic Root in Pigeons (*Columba livia*) Revealed With Biotinylated Dextran Amine.” Journal of Comparative Neurology 384: 517–536. 9259487 10.1002/(sici)1096-9861(19970811)384:4<517::aid-cne3>3.0.co;2-5

[cne70087-bib-0085] Wylie, D. R. , and N. A. Crowder . 2000. “Spatiotemporal Properties of Fast and Slow Neurons in the Pretectal Nucleus Lentiformis Mesencephali in Pigeons.” Journal of Neurophysiology 84: 2529–2540. 11067995 10.1152/jn.2000.84.5.2529

[cne70087-bib-0086] Wylie, D. R. , and B. J. Frost . 1999. “Responses of Neurons in the Nucleus of the Basal Optic Root to Translational and Rotational Flowfields.” Journal of Neurophysiology 81: 267–276. 9914287 10.1152/jn.1999.81.1.267

[cne70087-bib-0087] Wylie, D. R. , C. Gutierrez‐Ibanez , J. M. P. Pakan , and A. N. Iwaniuk . 2009. “The Optic Tectum of Birds: Mapping Our Way to Understanding Visual Processing.” Canadian Journal of Experimental Psychology/Revue Canadienne De Psychologie Expérimentale 63: 328–338. 20025392 10.1037/a0016826

[cne70087-bib-0090] Wylie, D. R. , A. H. Gaede , C. Gutiérrez‐Ibáñez , et al. 2023. “Topography of optic flow processing in olivo-cerebellar pathways in zebra finches (Taeniopygia guttata).” Journal of Comparative Neurology 531: 640–662. 36648211 10.1002/cne.25454

